# TiO_2_ nanoparticles promote tumor metastasis by eliciting pro-metastatic extracellular vesicles

**DOI:** 10.1186/s12951-023-02142-4

**Published:** 2023-10-27

**Authors:** Xupeng Mu, Kebang Hu, Anhui Wei, Jinping Bai, Li Feng, Jinlan Jiang

**Affiliations:** 1 Scientifc Research Center, China–Japan Union Hospital of Jilin University, Changchun, 130033 China; 2 Department of Urology, Lequn Branch, The First Hospital of Jilin University, Changchun, 130031 China; 3 Department of Regenerative Medicine, College of Pharmacy, Jilin University, Changchun, 130021 China; 4 Department of Chronic Disease, Jilin Province FAW General Hospital, Changchun, 130013 China; 5 Department of Radiation Oncology, China–Japan Union Hospital of Jilin University, Changchun, 130033 China

**Keywords:** Nanoparticles, Extracellular vesicles, Angiogenesis, Vascular permeability, Tumor metastasis

## Abstract

The development of nanotechnology has provided numerous possibilities for the diagnosis and treatment of cancer. Paradoxically, some in vivo experimental studies have also shown that nanoparticles (NPs) could promote tumor progression, but the specific mechanism is not yet clear. Primary tumors can release extracellular vesicles (EVs) which can promote the inoculation and growth of tumor cells that have metastasized to distant organs. So, whether nanomaterials can promote tumor progression through tumor-derived EVs deserves further research. Here, we showed that TiO_2_ NPs, widely used in nanomedicine, could trigger tumor-derived EVs with enhanced pro-metastatic capacity in vitro and in vivo. Mechanically, miR-301a-3p derived from NPs-elicited EVs could be delivered into vascular endothelial cells, which inhibited the expression of VEGFR2 and VE-cadherin by targeting S1PR1, leading to disrupt the tight junctions of vascular endothelial cells, thus to promote vascular permeability and angiogenesis, and induce the formation of pre-metastasis niches in vivo. This study emphasizes that it is urgent to consider the effect of NPs on EVs under long-term use conditions when designing nanodrugs for cancer treatment.

**Graphical Abstract:**

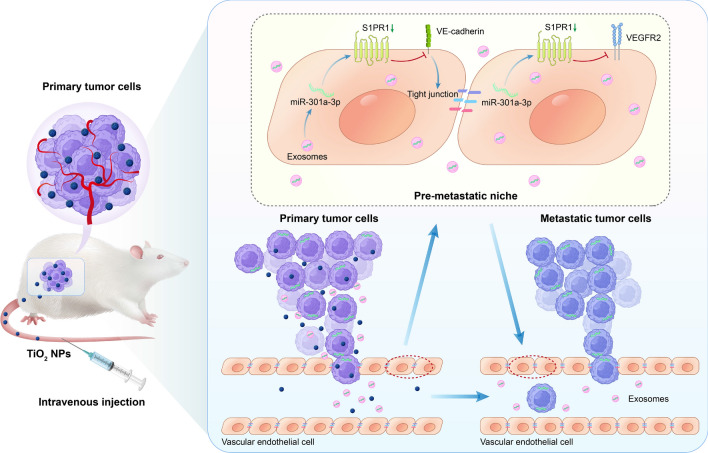

**Supplementary Information:**

The online version contains supplementary material available at 10.1186/s12951-023-02142-4.

## Introduction

Nanodrugs are being developed to treat various diseases, especially for cancers. However, inadvertent or accidental health effects must be considered. Although most nanodrugs used to treat cancer focus on the treatment of primary tumors, nevertheless, their impacts on tumor metastasis have not been fully studied or understood. To date, several reports have documented the side effects of NPs in promoting metastasis in vivo. It was found that carbon nanotubes (CNTs) could cause tumor lesions in the lung and pleura upon pulmonary exposure, which was related to local and systemic inflammation [1]. Previous studies have also reported that sub-acute lung exposure to SWCNTs (single-walled carbon nanotubes) could attract pre-existing MDSCs to enhance transforming growth factors beta (TGF-β) production, and then induced the growth of lung cancer in vivo [2]. Meanwhile, pulmonary CNTs have been proved to promote tumor metastasis by promoting the local formation of pro-metastatic niches. Increased expression of COX-2, CXCL2, MMP9, S100A9, and FN1 in CNT-exposed lung tissue established an acceptable microenvironment, including enhanced inflammation, angiogenesis, and immune suppression to form metastasis sites [3]. More recently, another new mechanism of NPs promoting tumor metastasis has been found. Leong et al. reported that nanomaterials (TiO_2_, Au, Ag, and SiO_2_ NPs) could promote intravasation of surviving cancer cells into the surrounding vasculature and subsequently extravasation, increasing the extent of existing metastasis and promoting the appearance of new metastatic sites by increasing endothelial leakiness in vivo [4]. In general, the above studies suggest that the ability of some NPs to promote pro-metastasis may include creating a microenvironment for tumor metastasis, or promoting invasion of cancer cells in primary tumors and extravasation to secondary metastatic sites. However, up to now, the exact mechanisms by which some NPs induced tumor metastasis have not been determined, prompting researchers to continue in-depth research.

It has been reported that extracellular vesicles (EVs) secreted by primary tumors could regulate the biology of distant organ niches by inducing vascular permeability, inflammation, and the recruitment of bone marrow progenitor cells in the distant organs to enhance the seeding and growth of metastatic cancer cells [5–10], but the effects of tumor derived EVs induced by nanomaterials on vascular permeability and tumor metastasis remain unclear. In this study, TiO_2_ NPs is selected as a research model, which is widely used in food products and as the core of nanodrug carrying materials. TiO_2_ NPs is often used as food additives to improve the color and taste of food, and can even be used as spices and spice carriers [11, 12]. This high usage efficiency may lead to higher levels of NPs in cancer patients.

**Fig. 1:**
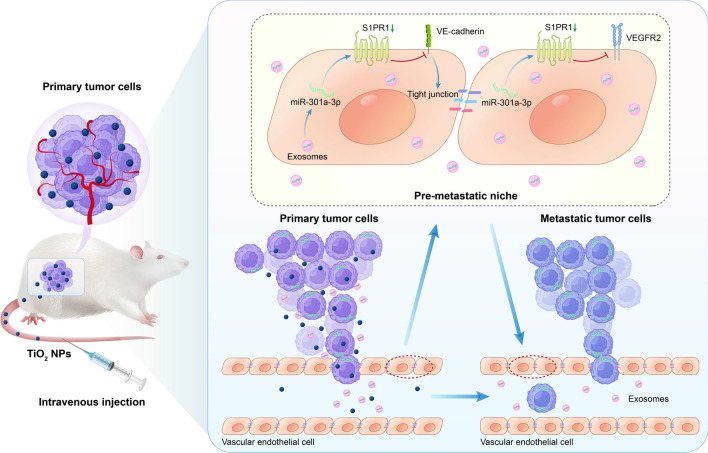
TiO_2_ NPs could promote tumor metastasis by eliciting pro-metastatic extracellular vesicles. TiO_2_ NPs could trigger tumor-derived EVs with enhanced pro-metastatic capacity in vivo. Mechanically, miR-301a-3p derived from NPs-elicited EVs could be delivered into vascular endothelial cells, which inhibited the expression of VEGFR2 and VE-cadherin by targeting S1PR1, leading to disrupt the tight junctions of vascular endothelial cells, thus to promote vascular permeability and angiogenesis, and induce the formation of pre-metastasis niches in vivo

Herein, we systematically investigated the effects of TiO_2_ NPs on the release, properties, and pro-metastatic potential of tumor derived EVs. So far, there have been no relevant studies on the effect of NPs on the secretion of tumor EVs. We identified that NPs-induced tumor-derived miR-301a-3p could be transferred to vascular endothelial cells and thereby promote vascular permeability and angiogenesis by targeting S1PR1, which destroyed the tight junctions of vascular endothelial cells and then induced the formation of pre-metastatic niche in vivo (Fig. 1).

## Materials and methods

### Materials

TiO_2_ NPs were obtained from Deke Daojin Science (Beijing, China). The primary sizes and shapes of the NPs were observed by Hitachi H-7500 transmission electron microscope (TEM, Jeol, Japan).

### Cell cultures

Human umbilical vein endothelial cells (HUVECs) were freshly isolated from umbilical cords and cultured in endothelial cell growth medium (ECGM) (Promocell, Germany) supplemented with 5% FBS, 5 ng/mL epidermal growth factor, 10 ng/mL basic fibroblast growth factor, 0.5 ng/mL vascular endothelial growth factor, 20 ng/mL insulin-like growth factor, and 0.2 μg/mL hydrocortisone.

Human breast cancer cells MDA-MB-231-Luc (American Type Culture Collection) and MCF-7 (American Type Culture Collection) were cultured in DMEM supplemented with 10% fetal bovine serum (FBS) and 1% penicillin–streptomycin. All cells were cultured according to the instructions.

### EVs isolation and characterization

EVs were isolated from tumor cells-derived conditioned media by sequential ultracentrifugation. MDA-MB-231 cells were cultured in DMEM supplemented with 10% EV-depleted FBS for 48 h. Then the conditioned media were collected, centrifuged at 500*g* for 5 min, 2000*g* for 10 min, and 16,800*g* for 20 min at 4 °C to remove dead cells and debris. The supernatants were passed through a 0.22 μm filter device (Merck, Germany), and then ultracentrifuged at 130,000*g* for 70 min at 4 °C. The pellets were washed with PBS, followed by ultracentrifugation at 130,000*g* for 70 min again. The resulting EVs were dissolved in PBS and stored at − 80 °C.

The prepared EVs were confirmed using transmission electron microscopy (TEM) and western blot. For TEM, purified EVs were placed on the carbon-coated 200 mesh grids to dry. The grids were then stained with 2% uranyl acetate for 30 s and observed using a Hitachi H-7500 TEM (Jeol, Japan).

The protein content of prepared EVs were measured using the BCA protein assay reagent kit (Beyotime, China). The size and concentration were measured by Nanoparticle Tracking Analysis (NTA) (Nanosight NS300; Malvern Instruments, UK). For EVs labeling, purified EVs were labeled with PKH67 Green dye (Merck, Germany) according to manufacturer's instructions. Then, the labeled EVs were collected by ultracentrifugation and resuspended in PBS.

### Immunofluorescence staining

Cells were fixed with pre-cooled 4% paraformaldehyde for 10 min and washed with PBS. The cells were then incubated with Triton X-100, and blocking buffer in PBS containing 5% BSA was added for 30 min. Subsequently, the cells were probed overnight with primary antibodies, followed by 1 h incubation with Alexa Fluor-conjugated secondary antibody. The following primary antibodies were used: CD31 (Proteintech, 11,265-1-AP, 1:200 dilution), VMF (Proteintech, 27,186-1-AP, 1:50 dilution), VE-cadherin (CST, 2158S, 1:1000 dilution), ZO-1 (Cell Signaling, #8193, 1:1000 dilution), S1PR1 (Proteintech, 55,133-1-AP, 1:200 dilution), LC3 (Proteintech, 14,600-1-AP, 1:500 dilution). DAPI (Beyotime, China) was used to stain the nuclei without light for 15 min. To visualize the actin filaments, cells were fixed, permeabilized and stained with TRITC phalloidin (Yeasen, China). The stained samples were observed by a confocal laser scanning microscope (IX5-RFACA; Olympus, Japan).

### RNA interference and plasmids

The miR-301a-3p inhibitor, mimics and negative control (NC) were synthesized by GenePharma (Shanghai, China). The sequences of inhibitor and miRNA mimics referred above were listed in Additional file 1: Table S1. The vectors overexpressing miR-301a-3p and inhibiting miR-301a-3p were constructed and produced. The cells including 293 T cells, HUVECs, and MDA-MB-231 cells were transfected above mentioned plasmids with Lipofectamine™ 3000 (Invitrogen, USA) and were used for further experiments 24 h–48 h, respectively.

### Quantitative RT-PCR

Total RNAs were extracted from cells or EVs using Trizol reagent (Invitrogen, USA) according to the manufacturer’s instruction. Thereafter, extracted RNA was reverse transcribed by using PrimeScript RT Master Mix kit for cDNA (TaKaRa, Japan). qRT-PCR was then performed by using SYBR Green PCR Master Mix (TaKaRa, Japan) on Applied Bio-systems 7500 Fast Real-Time RCR System (Applied Biosystems, USA). Each sample was performed in triplicate and the relative transcription levels of target genes were normalized to GAPDH or U6 (for miRNA). The sequences of all indicated primers are presented in Additional file 1: Table S1.

### Western blot assay

The cells and EVs lysates were extracted in RIPA lysis buffer (Beyotime, China). The protein content in the supernatant was quantified by BCA protein assay kit (Beyotime, China). Subsequently, equal amounts of denatured proteins were subjected to SDS-PAGE and transferred onto nitrocellulose membranes (Millipore, USA). After blocking with 5% nonfat dry milk for 1 h, the membranes were probed overnight at 4 °C with diluted primary antibodies. The following primary antibodies were used: Alix (Proteintech, 12,422-1-AP, 1:1000 dilution), CD9 (Proteintech, 20,597-1-AP, 1:1000 dilution), S1PR1 (Proteintech, 55,133-1-AP, 1:2000 dilution), LC3 (Proteintech, 14,600-1-AP, 1:2500 dilution), β-actin (Proteintech, 20,536-1-AP, 1:1000 dilution). After washing with TBST buffer, the membranes were further reprobed with IRDye-conjugated secondary antibodies (1:5000) for 1 h at room temperature. The membranes were analyzed using an Odyssey infrared imaging system (LI-COR Biosciences, USA).

### Cell viability assay

Cell viability was determined by the CCK-8 assay (Beyotime, China) according to the manufacturer’s instructions. Briefly, 5 × 10^3^ cells were seeded in 96-well plates and treated with EVs of different concentrations. After 24 h, 10 μl CCK-8 solution was supplemented to each well for another 2 h and absorbance at 450 nm was measured using a microplate reader (ELx-800; BioTek Instruments, USA).

### Transwell permeability assay

The integrity of endothelial cell barrier was determined by quantifying the amount of FITC-dextran (MW = 70 kDa, Merck, Germany) that passed through the endothelial monolayers. Briefly, 2 × 10^4^ HUVECs were grown on the top well of the transwell filters (0.4 μm pore; Corning Costar, USA) until the confluent monolayer was obtained. The cells were exposed to EVs (20 µg/mL) for 1 h, and then FITC-dextran (1 mg/mL) was supplemented. After EVs treatment, the FITC-dextran could be transferred to the lower compartment of transwell. FITC fluorescence signals were measured using a microplate reader (ELx-800; BioTek Instruments, USA) at 520 nm. The fluorescence signal of the treatment groups was normalized to the control group, and the leakage degree was given.

Transendothelial invasion assay was performed to study the effects of endothelial cell leakage on tumor cell migration. 2 × 10^4^ HUVECs were first seeded on the upper well of the transwell insert (8 μm pore; Corning Costar, USA) until confluent. Then, the HUVECs were treated with EVs (20 µg/mL) for 1 h. Thereafter, 1 × 10^4^ Cell-Tracker Green labeled MDA-MB-231 cells, MCF-7 cells, or Cell-Tracker Red labeled A549 cells resuspended in serum-free media were placed inside the upper chamber, respectively. Complete DMEM media containing 10% FBS was placed into the bottom chamber. After 24 h, non-migrated cells in the upper chamber of transwell inserts were removed with cotton buds and washed with PBS. Then, the tumor cells that invaded through HUVEC monolayers and stuck to the lower surface of the membrane were fixed with 4% paraformaldehyde and calculated under a inverted fluorescence microscope (IXplore Pro; Olympus, Japan) at least three random visual fields per well.

### Angiogenesis assay

For tube formation assay, matrigel matrix (100 μL/well) was plated in 48-well plate and incubated at 37 °C for 30 min to allow the matrigel to polymerize. The EVs-treated HUVECs were seeded on the matrigel-coated wells and then incubated for 6 h–12 h. The tube formation ability was determined by measuring the number of tubes with inverted microscope (IXplore Pro; Olympus, Japan).

For aortic ring assay, thoracic aortas excised from 4- to 8-week-old mice were removed excessive fat and cut into 1–1.5 mm long cross sections. Matrigel matrix (100 μL/well) was plated in 48-well plate and incubated at 37 °C for 30 min to facilitate matrigel polymerization. Then, rings were gently placed onto the polymerized matrigel layer and covered with an additional matrigel (50 μL) for 30 min at 37 °C. Subsequently, ECGM medium (Promocell, Germany) was added to each well. After 24 h incubation, the aortic rings were incubated with ECGM conditioned medium, which included EVs derived from MDA-MB-231 cells. And the media was replaced every 2 days. The number of sprouts was observed on day 3–5 with inverted microscope and quantified by counting all sprouts from one ring.

### Microarray analysis

The miRNA expressions of EVs derived MDA-MB-231 cells, TiO_2_ treated MDA-MB-231 cells were carried out in Illumina NextSeq 500 (Illumina, USA). After extracting and quantifying total RNA samples, the library was constructed, and the quality of the library was determined using Agilent 2100 Bioanalyzer. Sequencing libraries of different samples were mixed, and single-stranded DNA was generated by denaturing with 0.1 M NaOH. Sequencing was performed for 50 cycles using a Illumina NextSeq 500 sequencer. Then, we used miRDeep2 software to quantify known miRNAs and predict new miRNAs for all trimmed reads. Based on CPM standardized miRNAs, the R software edgeR was used to perform differential expression calculations and screen for differential miRNAs. The top differential miRNA target genes were statistically analyzed, and the GO and path analysis of the target genes were performed.

### Luciferase activity assay

The wild-type (wt) and mutant (mt) 3′-UTR segments of S1PR1 were inserted into the luciferase reporter plasmid psiCHECK-2 and amplified, respectively. Co-transfections of S1PR1 3′-UTR plasmids with miR-301a-3p mimics (50 nM) into 293 T cells or HUVECs were accomplished by using Lipofectamine 3000 reagent (Merck, Germany). Cell lysates were harvested 48 h after transfection, and luciferase activity was examined by a dual luciferase reporter assay kit. All assays were performed in triplicate and each experiment was repeated three times.

### Animal models

Immune-deficient NOD SCID mice (female, 4 weeks old) were purchased from the Vital River Company (Beijing, China). All animal experiments were performed in accordance with the Guidelines for Care and Use of Laboratory Animals of Jilin University and approved by the Animal Ethics Committee of Jilin University (Changchun, China). MDA-MB-231-Luc cells (5 × 10^6^) were suspended in 100 μL PBS containing matrigel matrix (5 mg/mL) and transplanted into the left fourth abdominal fat pad at the base of the nipple of the mouse. When the subcutaneous xenograft tumor reached ~ 100 mm^3^, the tumor-bearing mice were anaesthetized and injected with 5 µg of EVs in PBS or TiO_2_ NPs via tail vein every other day for a total of ten treatments. After 3 weeks, the mice were administered 15 μg of luciferin intraperitoneally per gram of body weight. And the bioluminescence intensity was observed with the IVIS Spectrum In Vivo Imaging System (PerkinElmer, USA). Then the tissue of mice were harvested for bioluminescence imaging and histology analysis right after being intraperitoneally injected with luciferin.

For in vivo vascular permeability analysis, mice were anaesthetized and injected with 5 µg of EVs in PBS via tail vein every other day for a total of ten treatments. Then 100 μL FITC-dextran (100 mg/kg) was intravenously injected into the tail vein of nude mice. After 2 h, the excessive dye was removed by the transcardiac perfusion, and the lungs of mice were taken for examination. For tail vein metastasis assay, 1 × 10^5^ MDA-MB-231 cells were injected into the tail vein of mice one day after the last EVs administration. After 2 weeks, mice injected with MDA-MB-231 cells were sacrificed and lungs were used to quantify metastatic tumor burden. To evaluate the metastasis, lungs were stained with hematoxylin/eosin (HE) and screened for metastatic nodules. Meanwhile, immunohistochemical staining for endothelial cell marker CD31, and tight junction protein ZO-1, was applied to evaluate the extent of vascular damage caused by EVs treatment.

### Statistical analysis

Statistical analyses were performed using GraphPad Prism. The statistical significance was ascertained using one-way ANOVA. Quantitative values of all experiments were expressed as the mean ± SD. *P* < 0.05 was considered significantly. Statistical significance of the data was summarized as follows: *P < 0.05, **P < 0.01, ***P < 0.001.

## Results

### TiO_2_ inorganic NPs promoted tumor metastasis in vivo

**Fig. 2:**
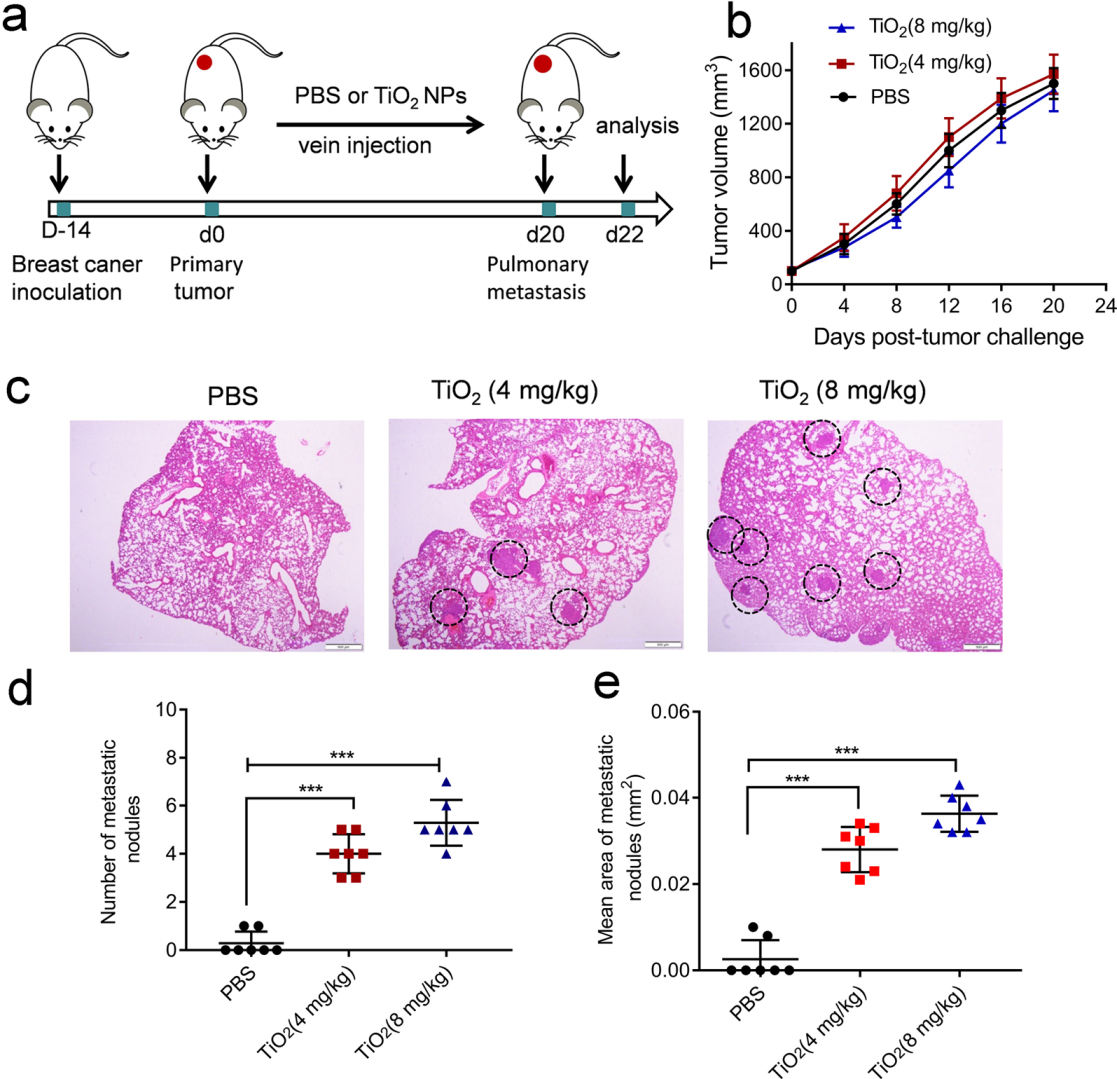
TiO_2_ NPs promoted tumor metastasis in vivo. **a** Schematic illustration of different treatments in tumor-bearing mice. **b** Volume of tumors treated with PBS or TiO_2_ during the period of 21 days post-tumor challenge. Data are presented as mean value ± SD (n = 6). **c** Hematoxylin/eosin (HE) images of lung sections of mice treated with PBS or TiO_2_. Scale bars = 500 µm**.** Data were quantified in (**d**) and (**e**). Number (**d**) and mean area (**e**) of pulmonary metastases in tumor-bearing mice. Data are shown as mean ± SD (n = 7). Statistical analysis was measured by one-way ANOVA

We firstly checked whether the TiO_2_ NPs could enhance tumor metastasis in mouse breast cancer models. The mice were administered either PBS or freshly prepared TiO_2_ NPs (60 nm; 4 mg/kg, 8 mg/kg) into tail vein every other day for a total of ten times before analysis (Fig. 2a). TiO_2_ NPs had almost no inhibitory effect on the growth of mice (Additional file 1: Fig. S1a). The weight, and volume of tumors have no significant changes compared with PBS group (Additional file 1: Fig. S1b and S2b). However, TiO_2_ NPs increased the incidence (Fig. 2c, d) and mean size of lung tumor metastases (Fig. 2e), in line with previous reports [4]. Together, these results indicated that TiO_2_ NPs may promote tumor metastasis in mouse models.

### TiO_2_ NPs induced EVs release from tumor cells

**Fig. 3:**
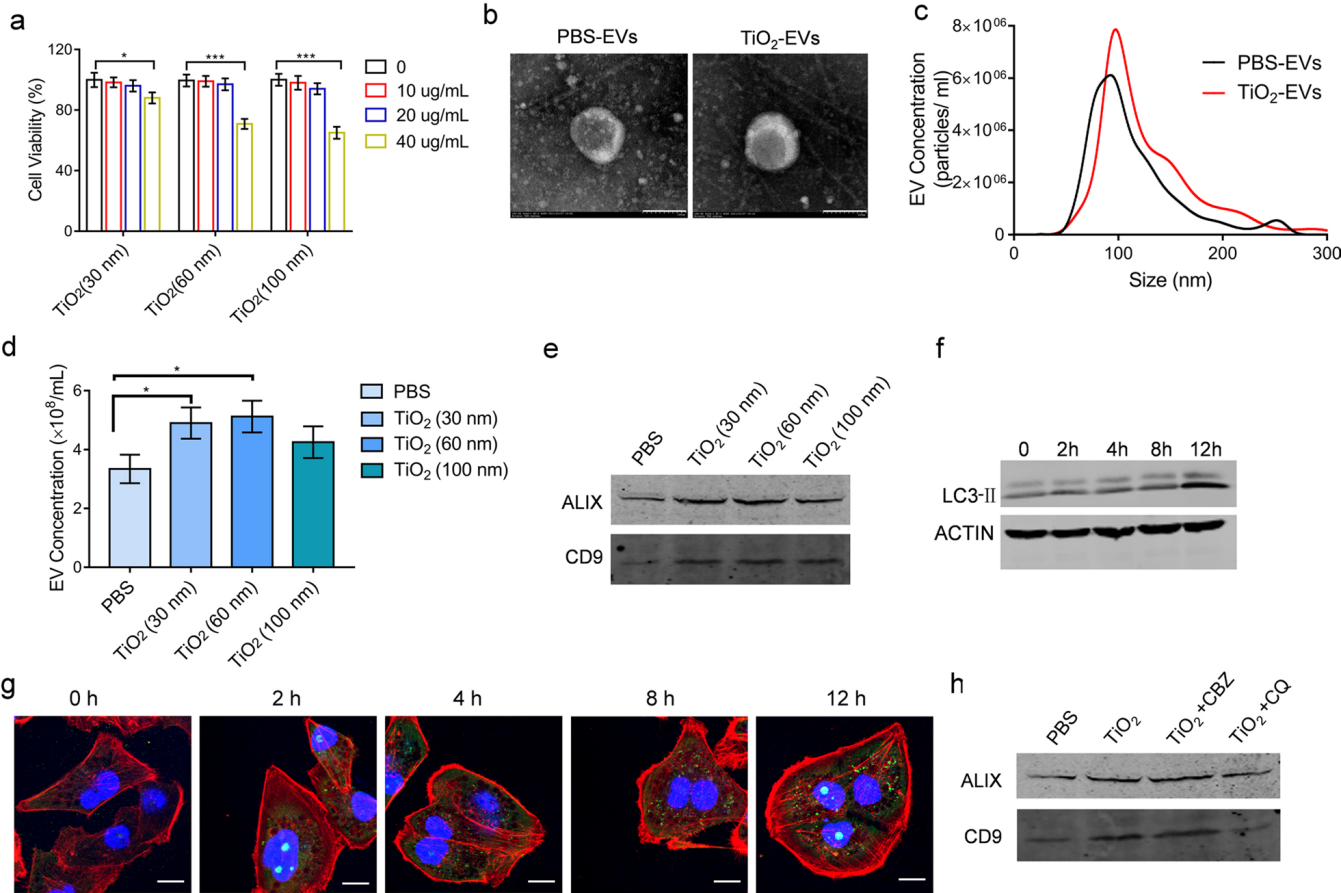
TiO_2_ NPs induced EVs release from tumor cells. **a** Relative survival rate of MDA-MB-231 cells treated with TiO_2_ NPs of different concentrations using CCK8 assay. Data are shown as mean ± SD (n = 3). **b** TEM images of PBS-EVs and TiO_2_-EVs isolated from cell supernatants after PBS or TiO_2_ treatment. Scale bars = 100 nm. **c** Concentration and size distribution of EVs treated as indicated, determined by NTA. **d** Concentrations of EVs isolated from cell supernatants treated with PBS or TiO_2_ NPs of different concentrations. Data are shown as mean ± SD (n = 3). **e** Western blotting analysis of CD9 and ALIX in tumor-derived EVs. **f** Western blotting analysis of LC3-II expression in tumor cells treated with TiO_2_ NPs for different time intervals. **g** Immunofluorescence analysis of LC3-II expression in tumor cells after treatment with TiO_2_ NPs for different time intervals. Scale bars = 10 μm. Nuclei, Blue (DAPI); LC3, Green; Phalloidin, Red. **h** Western blotting analysis of CD9 and ALIX in tumor-derived EVs after treatment with TiO_2_ NPs in the presence or absence of CQ or CBZ. Statistical analysis in (**a**) and (**d**) was measured by one-way ANOVA

We firstly examined the effects of TiO_2_ NPs with different particle sizes (30 nm, 60 nm, 100 nm) on EVs release from tumor cells (Additional file 1: Fig. S2). The CCK8 results showed that TiO_2_ NPs with different particle sizes (20 µg/mL) had almost no inhibitory effects on the cell growth, after cells were treated with TiO_2_ NPs of different concentrations (0, 10, 20, 40 µg/mL) (Fig. 3a). We then used sequential ultracentrifugation to isolate EVs from cell culture conditioned media of cancer cells treated with either TiO_2_ NPs or PBS (TiO_2_-EVs and PBS-EVs, respectively). The resulting preparations (TiO_2_-EVs induced by TiO_2_ NPs with a particle size of 60 nm) were measured by both TEM (Fig. 3b) and NTA (Fig. 3c). The TEM results revealed that the EVs preparations presented a typical shallow cup-shaped mouth, with a mode size between 30 and 150 nm. As shown in Fig. 3c, the particle sizes of PBS-EVs and TiO_2_-EVs are 92.5 nm and 97.5 nm, respectively. And the diameter of NPs had little effects on the size of prepared EVs. The NTA analysis showed that compared with the control group, TiO_2_ NPs could significantly promote the secretion of EVs from equal amounts of cells (Fig. 3d). The EVs-associated proteins CD9, and ALIX were enriched in TiO_2_-EVs compared to PBS-EVs, as demonstrated by western blot assays (Fig. 3e). Meanwhile, among the three kinds of TiO_2_ NPs with different particle sizes (30 nm, 60 nm, 100 nm), compared with TiO_2_ NPs (100 nm), TiO_2_ NPs (30 nm, 60 nm) could significantly promote the secretion of EVs (Fig. 3e). Finally, we selected TiO_2_ NPs (60 nm) as our research target.

Autophagy is a highly regulated catabolic process involving clearance, degradation, or exocytosis of damaged cell components or toxic aggregates, and protecting cells from damage [13]. It was found that the exocrine secretion of EVs was related to the amplification of autophagy by the NPs introduced the endosome [14]. Then we sought to clarify the role of autophagy in the exocytosis of TiO_2_-EVs. Cytoplasmic LC3-I will remove a small segment of polypeptide and transform into autophagic membrane LC3-II during the formation of the phagophore, and therefore the LC3-II/I ratio can estimate the autophagy level [15]. After treating cells with TiO_2_ NPs (60 nm) for different time intervals, the ratio of LC3-II to LC3-I was evaluated. We observed that TiO_2_ NPs treatment gradually increased the expression of LC3-II in a time dependent manner (Fig. 3f). Consistently, immunofluorescence analysis showed that treatment with TiO_2_ NPs resulted in a significant increase of green fluorescent vacuoles (Fig. 3g), confirming that TiO_2_ NPs treatment participated in the formation of autophages. To determine whether autophagy was involved in the exocytosis of TiO_2_-EVs, we exposed MDA-MB-231 cells with TiO_2_ NPs in the presence or absence of autophagy inhibitor chloroquine (CQ), or autophagy inducer carbamazepine (CBZ). The western blotting results revealed that CQ significantly inhibited the exocytosis of TiO_2_-EVs, while CBZ enhanced the exocytosis of TiO_2_-EVs (Fig. 3h), indicating that autophagy mediated the exocytosis of TiO_2_-EVs.

### TiO_2_ NPs-induced EVs promoted tumor metastasis in vivo

**Fig. 4:**
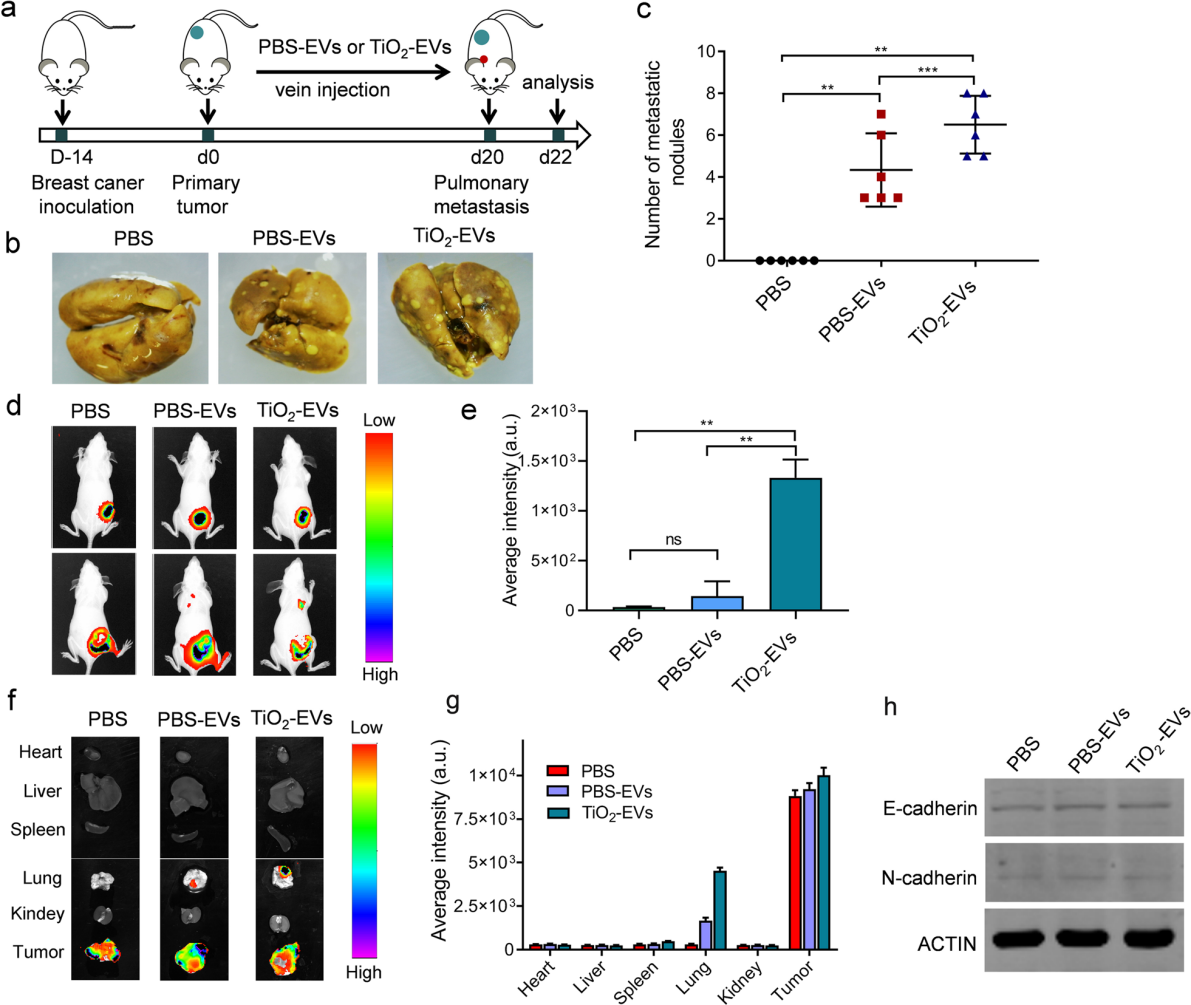
TiO_2_ NPs-induced EVs promoted tumor metastasis in vivo. **a** Schematic illustration of the establishment and experimental protocol in tumor-bearing mice. **b** Lungs metastases were visualized after fixed with paraformaldehyde containing 10% picric acid. White spots represent the metastatic tumors. **c** Number of pulmonary metastases in tumor-bearing mice treated with PBS-EVs or TiO_2_-EVs. Data are shown as mean ± SD (n = 6). Statistical analysis was measured by one-way ANOVA. **d** The representative bioluminescence images of mice were used to track the growth and metastasis of tumor cells after intravenous injection of PBS-EVs or TiO_2_-EVs. **e** Bioluminescence intensities of lung metastases after 3 weeks. **f**, **g** Ex vivo bioluminescence imaging (**f**) and intensities (**g**) of major organs after intravenous injection of PBS-EVs or TiO_2_-EVs. **h** Western blotting analysis of E-cadherin and N-cadherin in MDA-MB-231 cells treated with PBS-EVs or TiO_2_-EVs

Tumor-derived EVs could promote the metastasis of primary tumors by altering the properties of tumor microenvironment associated with pre-metastatic niche [5–10]. In order to measure the involvement of tumor-derived EVs induced by TiO_2_ NPs in tumor metastasis, we treated tumor-bearing mice with either TiO_2_-EVs or PBS-EVs via tail vein every other day for a total of ten treatments (Fig. 4a). In the lung metastases assay, TiO_2_-EVs increased the number of metastatic lung nodules compared to PBS-EVs (Fig. 4b, c). All the experimental groups exhibited similar rapid growth of the primary tumors. However, there is no significant difference in the volume and weight of the primary tumor between the experimental group and the control group (Additional file 1: Fig. S3a, b). After 3 weeks, bioluminescence signals could be detected in the chest region from mice receiving TiO_2_-EVs (5 µg) treatment (Fig. 4d), which may be caused by the metastasis of breast cancer cells to the lung region. The main organs were then collected for bioluminescence to track the metastasis. There were significant lung metastases in the TiO_2_-EVs-treated group compared with the PBS-EVs group (Fig. 4f). Furthermore, the bioluminescence intensities showed that compared with PBS group and PBS-EVs group, the lung fluorescence of the TiO_2_-EVs-treated group was the highest (Fig. 4e, g). These results confirmed that TiO_2_-EVs treatment could facilitate metastasis of primary tumors.

To exclude the influence of other factors on tumor metastasis, we studied the effect of EVs on intrinsic migratory ability of tumor cells. We examined this possibility with various migration assays using MDA-MB-231 cells that were exposed to TiO_2_-EVs or PBS-EVs. We did not find any significant changes in migration ability or epithelial-mesenchymal transition (EMT) markers after 24 h of EVs treatment (Additional file 1: Fig. S4a, b, and Fig. 4h).

### TiO_2_ NPs-induced EVs facilitated vascular leakiness and angiogenesis

**Fig. 5:**
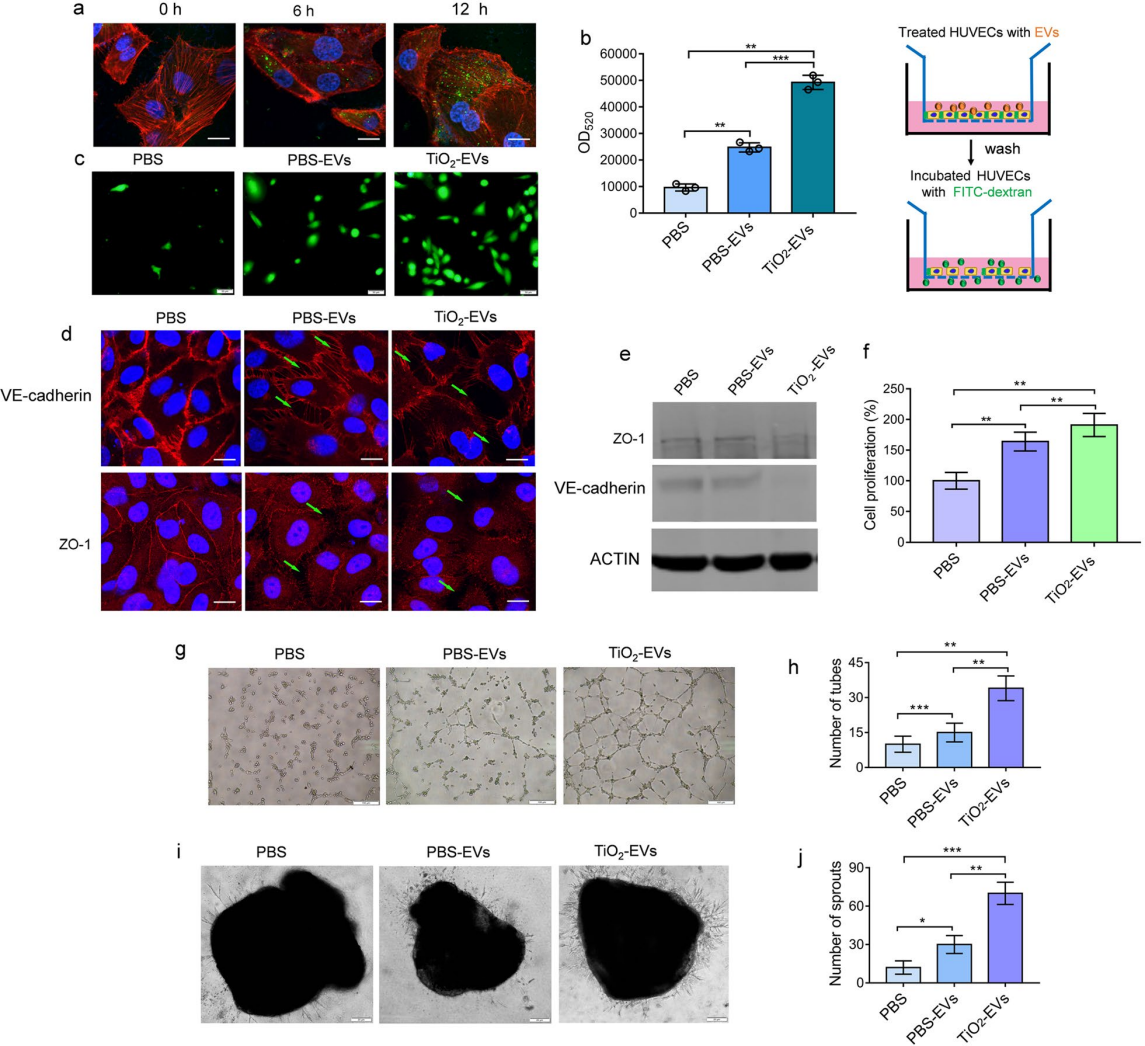
TiO_2_ NPs-induced EVs facilitated vascular leakiness and angiogenesis. **a** EVs was internalized into HUVECs. Time-dependent increase of fluorescence signal intensity was observed after incubation PKH67 labeled EVs with HUVECs. Scale bars = 10 μm. Nuclei, Blue (DAPI); PKH67 labeled EVs, Green; Phalloidin, Red. **b** Increased fluorescence intensity of FITC-dextran was observed following exposure of HUVECs to EVs for 1 h. Data are shown as mean ± SD (n = 3). **c** Cell-Tracker Green labeled MDA-MB-231 cells migrated through HUVECs monolayers were observed under fluorescent microscopy following exposure of HUVECs to EVs for 1 h. Scale bars = 50 μm. **d** PBS-EVs or TiO_2_-EVs exposure on HUVECs monolayers resulted in the disruption of VE-cadherin and ZO-1. Scale bars = 10 μm. Nuclei, Blue (DAPI); VE-cadherin or ZO-1, Red. (**e)** Western blotting analysis of VE-cadherin and ZO-1 in HUVECs that were treated with PBS-EVs or TiO_2_-EVs. **f** The proliferative capacity induced by PBS-EVs or TiO_2_-EVs using CCK8 assay. Data are shown as mean ± SD (n = 3). **g**, **h** Effects of PBS-EVs or TiO_2_-EVs on tube formation ability of HUVECs by tube formation assay. Scale bar = 100 µm. Data are shown as mean ± SD (n = 3). **i**, **j** Effects of PBS-EVs or TiO_2_-EVs on vascular outgrowth of aortic rings which was quantified by counting all sprouts from one ring. Scale bar = 20 µm. Data are shown as mean ± SD (n = 3). Statistical analysis in (**b**, **f**, **h** and **j**) was measured by one-way ANOVA

Tumor metastasis is a mixed etiology, the core of which is the invasion and infiltration of shed tumor cells through ruptured blood vessels. It is reported recently that tumor-derived EVs could regulate pre-metastatic niche formation by inducing angiogenesis and vascular permeability [16]. Then we intended to explore the impact of TiO_2_-EVs on endothelial cells. We firstly isolated human umbilical vein endothelial cells (HUVECs) from umbilical cords and cultured in endothelial cell growth medium (ECGM). The separated HUVECs grew and arranged like cobblestones. The expression of Von Willebrand factor (VWF) and platelet endothelial cell adhesion molecule-1 (CD31) was positive in separated HUVECs by immunofluorescence analysis (Additional file 1: Fig. S5). HUVECs were incubated with PKH67-labeled EVs derived from MDA-MB-231 cells for 12 h, PKH67 lipid dye could be observed in the incubated HUVECs and fluorescence signal intensity increased with time (Fig. 5a). The results suggested that EVs could be transferred into HUVECs.

In vitro permeability assay were firstly performed to detect whether TiO_2_-EVs could regulate vascular permeability. HUVECs treated with TiO_2_-EVs showed increased leakiness by measuring the fluorescence intensity of FITC-labeled dextran (MW = 70 kDa) across the HUVECs layer after 1 h of exposure, as compared with those treated with PBS or PBS-EVs (Fig. 5b). If the resultant cell gaps are large enough after EVs treatment, tumor cells can easily invade through HUVECs layer and stuck to the lower surface of the membrane. HUVECs were treated with TiO_2_-EVs or PBS-EVs for 1 h, and then Cell-Tracker Green pre-labeled MDA-MB-231 cells were added. 24 h later, the PBS-EVs had almost no or some sparsely MDA-MB-231 cells which could invade through HUVECs monolayer, in contrast, the large intercellular space of TiO_2_-EVs could induce more green-labeled MDA-MB-231 cells to invade through HUVECs layer (Fig. 5c). In addition, similar migration tendency were also observed in MCF-7 cells and A549 cells, which were labeled with Cell-Tracker Green and Cell-Tracker Red, respectively (Additional file 1: Fig. S6a, b, c).

Since the permeability of the endothelial cells layer is associated with its tight junction related proteins, tight junction proteins in endothelial cells were examined. Consistent with the above results, treatment with TiO_2_-EVs significantly destroyed the tight junction related proteins, including ZO-1, and VE-cadherin (Fig. 5d). The western blotting results also revealed that there was a marked reduction in both ZO-1 and VE-cadherin in HUVECs after treatment with TiO_2_-EVs, but not with PBS or PBS-EVs (Fig. 5e). The results indicated that TiO_2_-EVs could promote the increase of the permeability of endothelial cell neovascularization.

The formation of pre-metastatic niche in distant organs is closely related to angiogenesis. The migration of vascular endothelial cells is an important step of angiogenesis, compared to PBS-EVs, treatment with TiO_2_-EVs dramatically promoted the proliferation (Fig. 5f) and migration of HUVECs after incubating for 24 h (Additional file 1: Fig. S7a, b). Tube formation assay, aortic ring assay were then performed to detect whether TiO_2_-EVs was involved in regulating angiogenesis. As shown in Fig. 5g and h, compared with PBS or PBS-EVs groups, there was a significant increase in the microtube formation ability of HUVECs treated with TiO_2_-EVs. Meanwhile, thoracic aortas excised from mice were cut into 1–1.5 mm long cross sections and embedded in the matrigel. The number of extended microvessels in the matrix gel represents the ability of angiogenesis. The aortic ring assay revealed that the number of aortic rings increased significantly after treatment with TiO_2_-EVs (Fig. 5i, j). These results suggested that TiO_2_-EVs derived from tumor cells could disrupt the integrity of endothelial barriers and induce angiogenesis.

### S1PR1 was a functional target of miR-301a-3p from TiO_2_-induced EVs

**Fig. 6:**
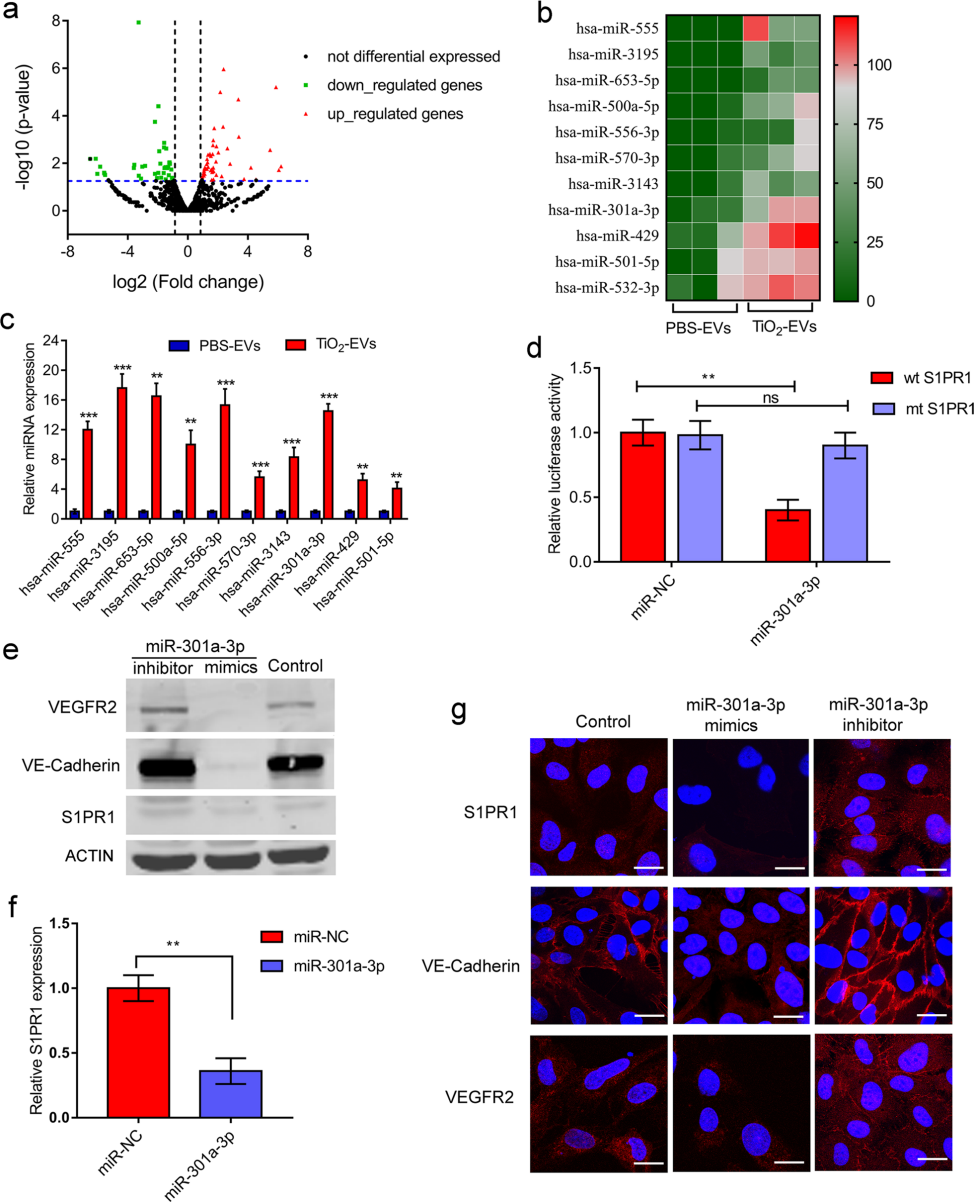
S1PR1 was a functional target of miR-301a-3p from TiO_2_-induced EVs in HUVECs. **a** Volcano plot of the DE-miRNAs. The black dots represent miRNAs that are not differentially expressed between PBS-EVs and TiO_2_-EVs, and the red dots and green dots represent the upregulated and downregulated miRNAs, respectively. **b** Heat map of the eleven upregulated miRNAs compared to PBS-EVs. **c** RT-PCR analysis of differentially expressed miRNAs in PBS-EVs and TiO_2_-EVs. Data are shown as mean ± SD (n = 3). **d** The relative luciferase activity of wild-type (wt) and mutant-type (mt) 3′-UTR of S1PR1 was determined. Data are shown as mean ± SD (n = 3). **e** Western blotting analysis of VE-cadherin, S1PR1, and VEGFR2. **f** RT-PCR analysis of S1PR1 expression in HUVECs which were treated with a miR-301a-3p mimic. Data are shown as mean ± SD (n = 3). **g** Immunofluorescence analysis of VE-cadherin, S1PR1, and VEGFR2. miR-301a-3p was overexpressed via a miR-301a-3p mimic or downregulated using a miR-301a-3p inhibitor in HUVECs, respectively. Scale bars = 10 μm. Nuclei, Blue (DAPI); S1PR1, VE-cadherin or VEGFR2, Red. Statistical analysis in (**c**, **d** and **f**) was measured by one-way ANOVA

The enhanced pro-metastatic ability of TiO_2_-induced EVs may depend on their components inside EVs. To explore the mechanisms of TiO_2_-EVs disrupting the integrity of vascular endothelial barriers and inducing angiogenesis, we performed miRNA microarray analysis of MDA-MB-231-derived TiO_2_-EVs and PBS-EVs to find potential metastasis-associated miRNAs, since microRNAs (miRNAs) from EVs play important roles in EVs-mediated effects. TiO_2_-EVs and PBS-EVs exhibited different miRNA compositions (Additional file 1: Fig. S8a, b). A total of 734 differentially expressed miRNAs were identified, of which 359 were upregulated and 375 were downregulated by TiO_2_-EVs compared to PBS-EVs (Additional file 1: Fig. S8b). A volcano plot of these differential miRNAs was provided in Fig. 6a. Gene Ontology (GO) functional annotation analysis were performed on these potential target genes, including biological process (BP), molecular function (MF) and cellular component (CC). As shown in Additional file 1: Fig. S9a–c, the enriched GO functions for target genes of the upregulated miRNAs included regulation of cellular process, developmental process, protein binding, transcription regulator activity. Meanwhile, KEGG signaling pathway enrichment analysis revealed significant differences in cancer related genes between PBS-EVs and TiO_2_-EVs (Additional file 1: Fig. S9d). The ten miRNAs with significant difference between TiO_2_-EVs and PBS-EVs were listed in Fig. 6b, and the differential expressions of these miRNAs were verified by quantitative PCR (Fig. 6c). Among the differently expressed miRNAs, miR-301a-3p was overexpressed in various cancers and could promote tumor development, metastasis, and drug resistance [17–20]. The data indicated that the expression level of miR-301a-3p in MDA-MB-231-derived TiO_2_-EVs was remarkably higher than that in PBS-EVs (Fig. 6c). However, the effects of EVs derived miR-301a-3p and the underlying mechanisms by which miR-301a-3p regulates endothelial cells still remain to be illustrated.

miRNA can bind to the 3'-UTR region of the target gene, inhibit mRNA translation or induce its degradation, thereby reducing the expression level of the target protein. The target genes of miR-301a-3p were predicted using TargetScan (http://www.targetscan.org/) and miRDB (http://mirdb.org/), and the results revealed that S1PR1 was a candidate target of miR-301a-3p (Additional file 1: Fig. S10). To further clarify whether miR-301a-3p could directly target S1PR1, wild-type (wt) and mutant (mt) 3′-UTR segments of S1PR1 were cloned into downstream of the luciferase reporter plasmids. After co-transfection with miR-301a-3p mimics in 293 T cells, the relative luciferase activity of wt 3′-UTR of S1PR1 was inhibited by about 60%, while the luciferase activity of mt 3′-UTR of S1PR1 was almost unchanged compared with that of the control group (Fig. 6d). Meanwhile, the relative luciferase activity measured in HUVECs was lower than that in 293 T cells, but the overall trend remained unchanged (Additional file 1: Fig. S11). Notably, the luciferase activities of wt S1PR1 3'-UTR could be suppressed by miR-301a-3p in 293 T cells and HUVECs. Moreover, ectopic overexpression of miR-301a-3p via a miR-301a-3p mimic could inhibit the expression of S1PR1 significantly, while downregulation of miR-301a-3p expression using a miR-301a-3p inhibitor dramatically increased the expression of S1PR1 (Fig. 6e, f). These results indicated that miR-301a-3p could directly bind to the 3'-UTR region of S1PR1 and further inhibit the expression of downstream related proteins.

It has been found that S1PR1 could inhibit angiogenesis and promote vascular stability mainly by regulating VE-cadherin and VEGFR2 [21]. After specific knockout of S1PR1 in endothelial cells, the distribution of VE-cadherin in the cell membrane was significantly reduced, resulting in loose connections between endothelial cells, which promoted the exchange of cells and substances inside and outside the blood vessels. Meanwhile, S1PR1 could antagonize the VEGF-VEGFR2 pathway, and inhibit the angiogenic effect of VEGF [22, 23]. We then explored whether miR-301a-3p could affect the cell membrane localization function of VE-cadherin after inhibiting the expression of S1PR1. Western blot results showed that overexpression of miR-301a-3p in endothelial cells significantly reduced the expression level of VE-cadherin while reducing S1PR1, indicating that the difference in S1PR1 expression could affect the expression of VE-cadherin in the cell membrane (Fig. 6e). The immunofluorescence analysis also showed that overexpression of miR-301a-3p in HUVECs resulted in loss of S1PR1 and the co-localization of VE-cadherin from junctions compared to the control group (Fig. 6g).

We then examined whether S1PR1 could antagonize the VEGF-VEGFR2 pathway. The immunofluorescence analysis and western blotting results were consistent with VE-cadherin protein. Compared with the control group, overexpression of miR-301a-3p in endothelial cells also could significantly reduce the expression of VEGFR2 (Fig. 6e, g). The results indicated that miR-301a-3p could inhibit the expression of VEGFR2 and VE-cadherin by directly targeting S1PR1.

### Tumor cells-secreted miR-301a-3p induced vascular leakiness and promoted tumor metastasis in vivo

**Fig. 7:**
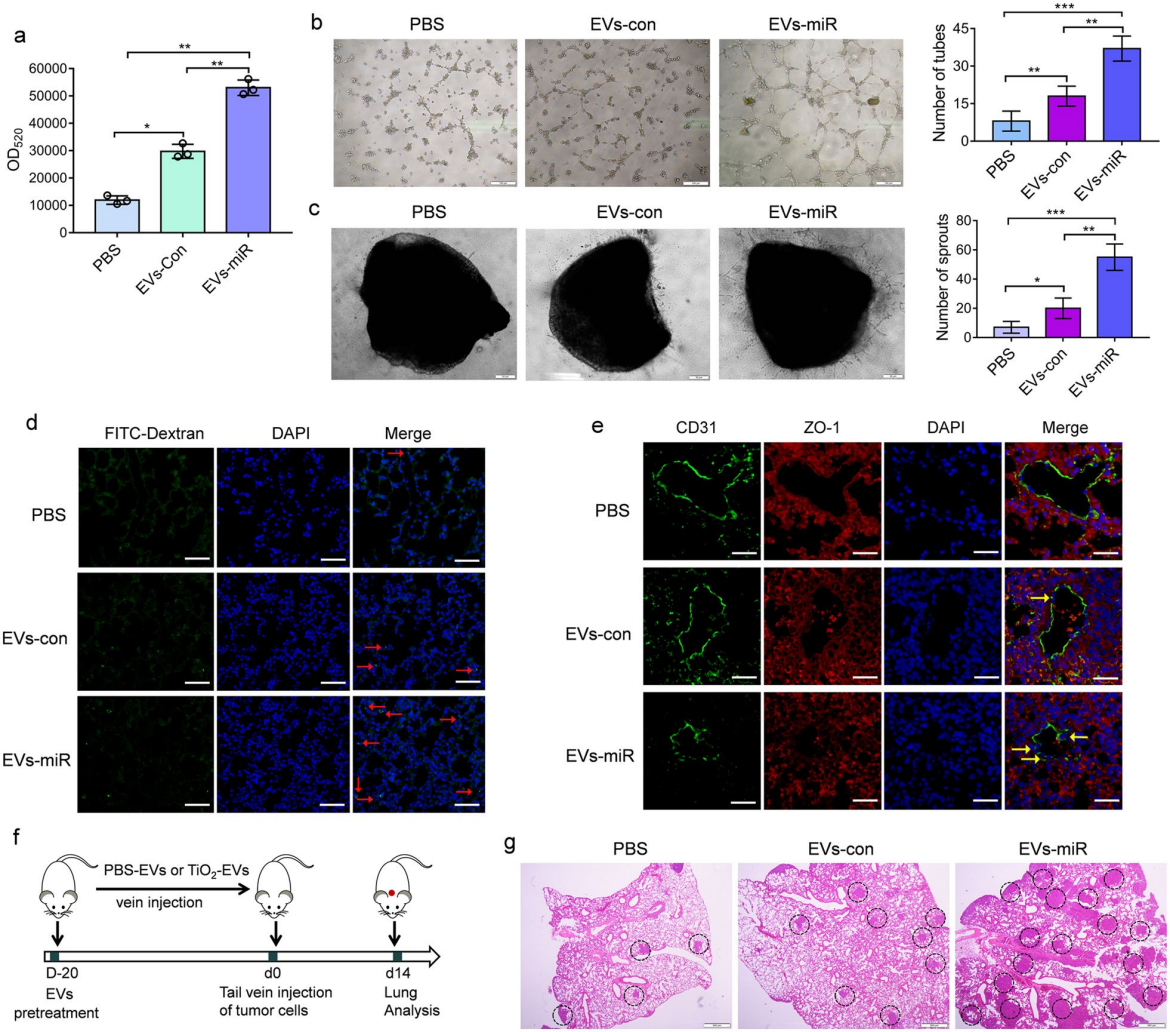
Tumor cells-secreted miR-301a-3p induced vascular leakiness and promoted tumor metastasis in vivo. **a** Increased fluorescence intensity of FITC-dextran was observed following exposure of HUVECs to EVs-miR or EVs-con for 1 h. Data are shown as mean ± SD (n = 3). **b** Effects of EVs-miR or EVs-con on tube formation ability of HUVECs by tube formation assay. Scale bar = 100 µm. Data are shown as mean ± SD (n = 3). **c** Effects of EVs-miR or EVs-con on vascular outgrowth of aortic rings which was quantified by counting all sprouts from one ring. Scale bar = 20 µm. Data are shown as mean ± SD (n = 3). **d** Mice were injected with EVs-miR or EVs-con for a total of ten treatments, and then the appearance of injected FITC-dextran was examined in lung. Scale bars = 50 μm. Nuclei, Blue (DAPI); FITC-dextran, Green. **e** Immunohistochemical staining for CD31, ZO-1, was applied to evaluate the extent of vascular damage caused by EVs-miR or EVs-con treatment. Scale bars = 50 μm. Nuclei, Blue (DAPI); CD31, Green; ZO-1, Red. **f** Schematic illustration of the establishment and experimental protocol for tail vein metastasis assay. **g** Representative hematoxylin/eosin (HE) images of lung sections of mice pre-treated with EVs-miR or EVs-con. Scale bars = 500 µm**.** Statistical analysis in **a**–**c** was measured by one-way ANOVA

It was shown that miRNA could be transferred from cells to cells through EVs [24]. Therefore, we investigated whether miR-301a-3p could be transferred to HUVECs through EVs. To investigate this issue, miR-301a-3p was over-expressed in MDA-MB-231 cells, and then EVs was extracted from condition media of MDA-MB-231/miR-301a-3p cells. RT-PCR showed that over-expression of miR-301a-3p in MDA-MB-231 cells led to the upregulation of EVs-derived miR-301a-3p, compared to control MDA-MB-231 cells (Additional file 1: Fig. S12). Then we detected whether MDA-MB-231/miR-301a-3p derived EVs (EVs-miR) could promote the migration of HUVECs. As shown in Additional file 1: Fig. S13, compared with the control group, HUVECs treated with MDA-MB-231/miR-301a-3p derived EVs (EVs-miR) which had high expression of miR-301a-3p dramatically promoted migration of HUVECs. Meanwhile, compared with control group (EVs-con), EVs from MDA-MB-231/miR-301a-3p (EVs-miR) obviously promoted vascular permeability (Fig. 7a), tube formation (Fig. 7b), and vascular outgrowth of aortic rings (Fig. 7c).

To evaluate whether EVs-derived miR-301a-3p plays a role in vascular permeability in pre-metastatic niche, EVs from MDA-MB-231 cells (EVs-con), MDA-MB-231/miR-301a-3p (EVs-miR) were injected into tail vein of nude mice every other day for a total of ten treatments, respectively. FITC-dextran (100 mg/kg) was injected into mice 2 h before they were killed. The fluorescence signals of section results demonstrated that EVs from MDA-MB-231/miR-301a-3p cells (EVs-miR), but not those from MDA-MB-231 cells (EVs-con), induced an obvious increase of signal in the lung, indicating that the highly expressed miR-301a-3p from EVs (EVs-miR) promoted the vascular permeability of the lung, and created conditions for new metastatic foci caused by tumor cell leakage (Fig. 7d). We carried out immunofluorescence staining on the blood vessels in the lung tissues of mice in each group, and observed that the expression level of ZO-1 and CD31 in the vascular endothelium of mice was significantly reduced when treated with EVs-miR (Fig. 7e). The HE results showed that EVs had no obvious toxic effect on other organs of mice (Additional file 1: Fig. S14).

We then investigated whether EVs pretreatment could increase metastasis opportunities in mice by intravenous injection of MDA-MB-231 cells. After 2 weeks, mice were sacrificed and lungs were removed for examination (Fig. 7f). In the lung colonization assay, The HE results revealed that EVs-miR treatment increased the number of metastatic lung nodules in mice (Fig. 7g).

## Discussion

Nanotechnology offers many possible applications in diagnosing and treating cancers. However, so far, several reports have documented pro-metastatic effects of NPs in vitro and in vivo. These studies lead us to further explore the causes of tumor metastasis caused by NPs. The potential participation of NPs-induced tumor-derived EVs in the process of tumor metastasis is currently unclear and may be challenging to explore. Initially, our data suggested that TiO_2_ NPs (30 nm, 60 nm) could significantly promote the secretion of EVs. Meanwhile, TiO_2_ NPs treatment gradually increased the expression of LC3-II in a time dependent manner.

The EVs derived from tumor cells are associated with tumor metastasis. They can promote carcinogenesis, proliferation, migration, invasion, immunosuppression, and angiogenesis, and formation of a pre-metastatic microenvironment. Increased angiogenesis/vascular permeability is important for the formation of tumor microenvironment and tumor metastasis [25]. Meanwhile, under the influence of various substances secreted by tumor cells, the permeability of new blood vessels is improved, making it easier for tumor cells to dissociate from the circulatory system and colonize at specific sites [26]. Intriguingly, our results showed that NPs induced tumor-derived EVs (TiO_2_-EVs) inhibited the expression of several tight junction proteins in HUVECs, including ZO-1 and VE-cadherin, both in vitro and in vivo. In vivo experiments, our data indicated that intravenous injection of TiO_2_-EVs increased the vascular permeability of the lungs. TiO_2_-EVs pre-treatment also resulted in a significant increase in tumor metastasis. There are many reasons why EVs promote tumor metastasis, but TiO_2_-EVs could promote tumor metastasis more effectively than PBS-EVs by disrupting vascular integrity and promoting tumor angiogenesis. MiR-301a was overexpressed in several types of tumors and its high expression was associated with an increased risk of recurrence [27, 28]. Our data further suggested that miR-301a-3p derived from NPs-elicited EVs could be delivered into endothelial cells. MiR-301a-3p could directly bind to the 3'-UTR segment of S1PR1 and affect the expression of S1PR1. And the decreased expression of S1PR1 affected the localization of the VE-cadherin in endothelial cells, which is also an important reason for the increased permeability of new blood vessels.

In conclusion, we demonstrated that miR-301a-3p derived from NPs-elicited EVs could directly inhibit the expression of S1PR1 in endothelial cells, and then regulate the expression of VEGFR2, VE-cadherin, consequently promoted vascular permeability and angiogenesis. This is the first demonstration that NPs can promote tumor metastasis by eliciting pro-metastatic extracellular vesicles. At the same time, the significance of EVs-derived miR-301a-3p for tumor metastasis may require further research. However, further exploration is needed to determine whether other nanomaterials besides TiO_2_ NPs can cause the same problem. This study prompts us to further investigate the effects of nanomaterials other than TiO_2_ NPs on the secretion of extracellular vesicles in tumor cells, and how to avoid this phenomenon.

### Supplementary Information

**Additional file 1:** **Figure S1.** TiO_2_ inorganic NPs promoted tumor metastasis in vivo. **a** Body weight of mice treated with PBS or TiO_2_ during the period of 21 days post-tumor challenge. **b** Tumor weight of mice treated with PBS or TiO_2_ during the period of 21 days post-tumor challenge. Data are presented as mean value ± SD (n = 6). Statistical analysis was measured by one-way ANOVA. **Figure S2.** TEM images of TiO_2_ NPs with different sizes. **a** 30 nm, **b** 60 nm. Scale bars = 100 nm. **c** 100 nm. Scale bars = 200 nm. **Figure S3.** Tumor volume (**a**) and tumor weight (**b**) of mice treated with PBS-EVs or TiO_2_-EVs during the period of 21 days post-tumor challenge. Data are presented as mean value ± SD (n = 6). Statistical analysis was measured by one-way ANOVA. **Figure S4.** The effects of PBS-EVs or TiO_2_-EVs on tumor migration ability. Wound healing assay (**a**) and transwell assay (**b**) were used to detect the effects of PBS-EVs or TiO_2_-EVs on tumor migration ability. Scale bars = 50 μm. Data are presented as mean value ± SD (n = 3). Statistical analysis was measured by one-way ANOVA. **Figure S5.** Immunofluorescence analysis of the expression of Von Willebrand factor (VWF) and platelet endothelial cell adhesion molecule-1 (CD31) in separated HUVECs. Scale bars = 20 μm. Nuclei, Blue (DAPI); CD31 or VWF, Red. **Figure S6.** Cell-Tracker Green labeled MCF-7 cells (**a**) and Cell-Tracker Red labeled A549 cells (**b**) migrated through HUVECs layers were observed under fluorescent microscopy following exposure of HUVECs to EVs for 1 h. Scale bars = 50 μm. **c** Experimental scheme for the migration assay. **Figure S7.** The effects of PBS-EVs or TiO_2_-EVs on HUVECs migration ability. Wound healing assay (**a**) and transwell assay (**b**) were used to detect the effects of PBS-EVs or TiO_2_-EVs on HUVECs migration ability. Scale bars = 50 μm. Data are presented as mean value ± SD (n = 3). Statistical analysis was measured by one-way ANOVA. **Figure S8. a** Cluster diagram of differential expression miRNA. Each row represented one miRNA, and each column represented one sample. The red dots and green dots represented the upregulated and downregulated miRNAs, respectively **b** Scatter Plot of the DE-miRNAs. The black dots represent miRNAs that are not differentially expressed between PBS-EVs and TiO_2_-EVs, and the red dots and green dots represent the upregulated and downregulated miRNAs, respectively. **Figure S9.** GO functions for the potential target genes of the top upregulated miRNAs were performed, including **a** Biological process (BP), **b** Cellular component (CC), **c** Molecular function (MF). **d** KEGG signaling pathway enrichment analysis were performed for significant differences in cancer related genes between PBS-EVs and TiO_2_-EVs. **Figure S10. a** Prediction of the binding sites of miR-301a-3p and 3′-UTR of S1PR1. **b** Construction of a reporter gene plasmid containing wild-type (wt) or mutant (mt) 3′-UTR of S1PR1 binding sites. **Figure S11.** The relative luciferase activity of wild-type (wt) and mutant-type (mt) 3′-UTR of S1PR1 was determined in HUVECs. miR-301a-3p inhibited the luciferase activity of reporter containing wild-type but not mutant 3′-UTR of S1PR1. Data are shown as mean ± SD (n = 3). Statistical analysis was measured by one-way ANOVA. **Figure S12.** RT-PCR analysis of miR-301a-3p expression in EVs derived from MDA-MB-231 cells which were treated with a miR-301a-3p mimic. Data are shown as mean ± SD (n = 3). Statistical analysis was measured by one-way ANOVA. **Figure S13.** Transwell assay were used to detect the effects of EVs-con or EVs-miR on HUVECs migration ability. Scale bars = 50 μm. Data are presented as mean value ± SD (n = 3). Statistical analysis was measured by one-way ANOVA. **Figure S14.** HE staining images of the major organs of mice from different treatment groups. The results indicated that the EVs had no obvious toxic effects on the organs of mice. Scale bar = 50 µm. **Figure S15.** The membrane images for all Western blotting reported in the main figures of this study. **Table S1.** The primers used in the mRNA/miRNA expression analysis experiment.

## Data Availability

The data of this study is available from the corresponding authors on reasonable request.
