# Supplementary material for: TiO2 nanoparticles promote tumor metastasis by eliciting pro-metastatic extracellular vesicles
Source: J Nanobiotechnology. 2023 Oct 27;21:392. doi: 10.1186/s12951-023-02142-4 (PMC10604521; doi:10.1186/s12951-023-02142-4)

**Supplementary Information**

**TiO_2_ nanoparticles promote tumor metastasis by eliciting pro-metastatic extracellular vesicles**

Xupeng Mu^1†^, Kebang Hu^2†^, Anhui Wei^3^, Jinping Bai^4^, Li Feng^5,*^ and Jinlan Jiang^1,*^

^1^Scientifc Research Center, China-Japan Union Hospital of Jilin University, Changchun 130033, China.

^2^Department of Urology, Lequn Branch, The First Hospital of Jilin University, Changchun,130031, China.

^3^Department of Regenerative Medicine, College of Pharmacy, Jilin University, Changchun 130021, China.

^4^Department of Chronic Disease, Jilin province FAW General Hospital, Changchun 130013, China.

^5^Department of Radiation Oncology, China-Japan Union Hospital of Jilin University, Changchun 130033, China.

†Xupeng Mu and Kebang Hu are contributed equally to this work.

*Correspondence:

Li Feng f_l@jlu.edu.cn

Jinlan Jiang  [jiangjinlan@jlu.edu.cn](mailto:jiangjinlan@jlu.edu.cn)

Full list of author information is available at the end of the article

**
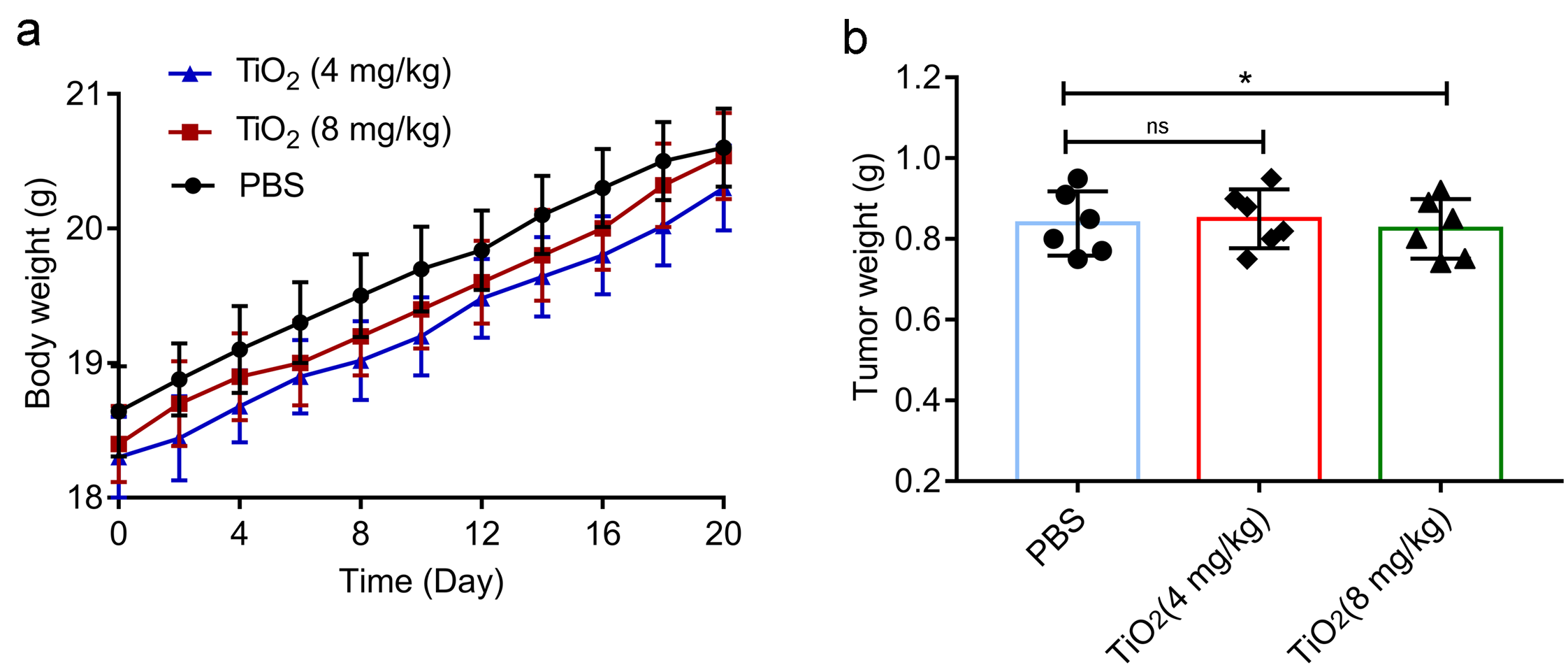
**

**Fig.S1** TiO_2_ inorganic NPs promoted tumor metastasis in vivo. **(a)** Body weight of mice treated with PBS or TiO_2_ during the period of 21 days post-tumor challenge. **(b)** Tumor weight of mice treated with PBS or TiO_2_ during the period of 21 days post-tumor challenge. Data are presented as mean value ± SD (n=6). Statistical analysis was measured by one-way ANOVA.

**
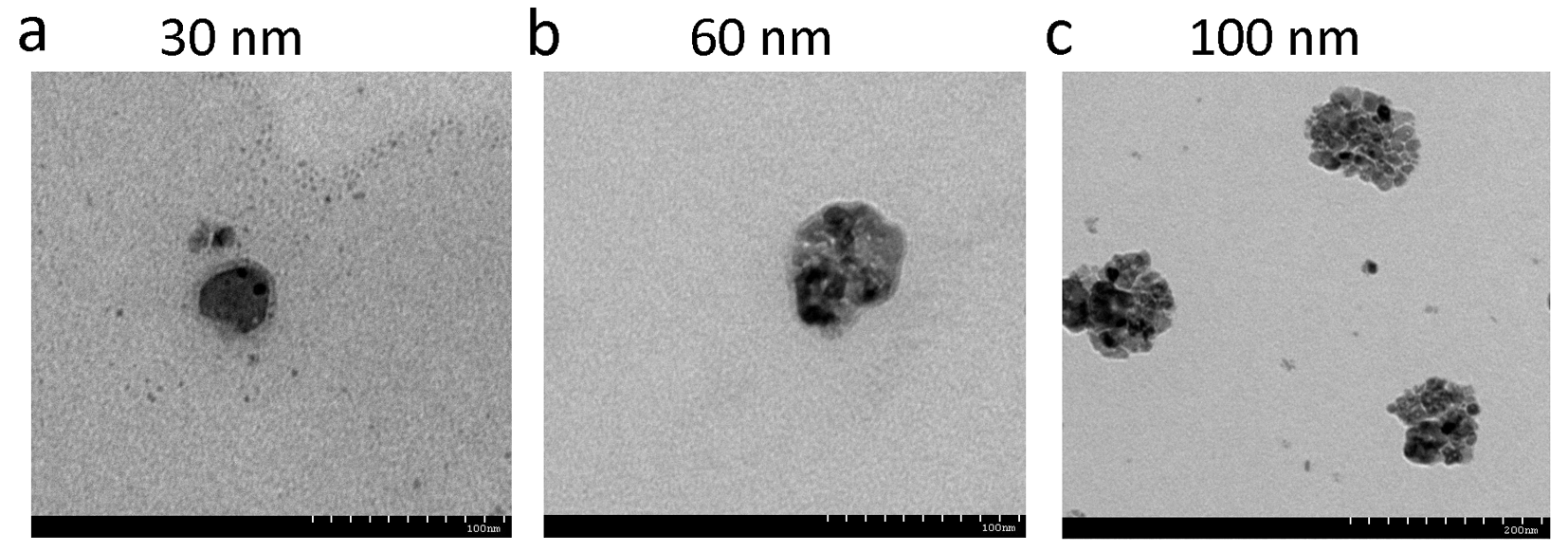
**

**Fig.S2** TEM images of TiO_2_ NPs with different sizes. **(a)** 30 nm, **(b)** 60 nm. Scale bars=100 nm. **(c)** 100 nm. Scale bars=200 nm.


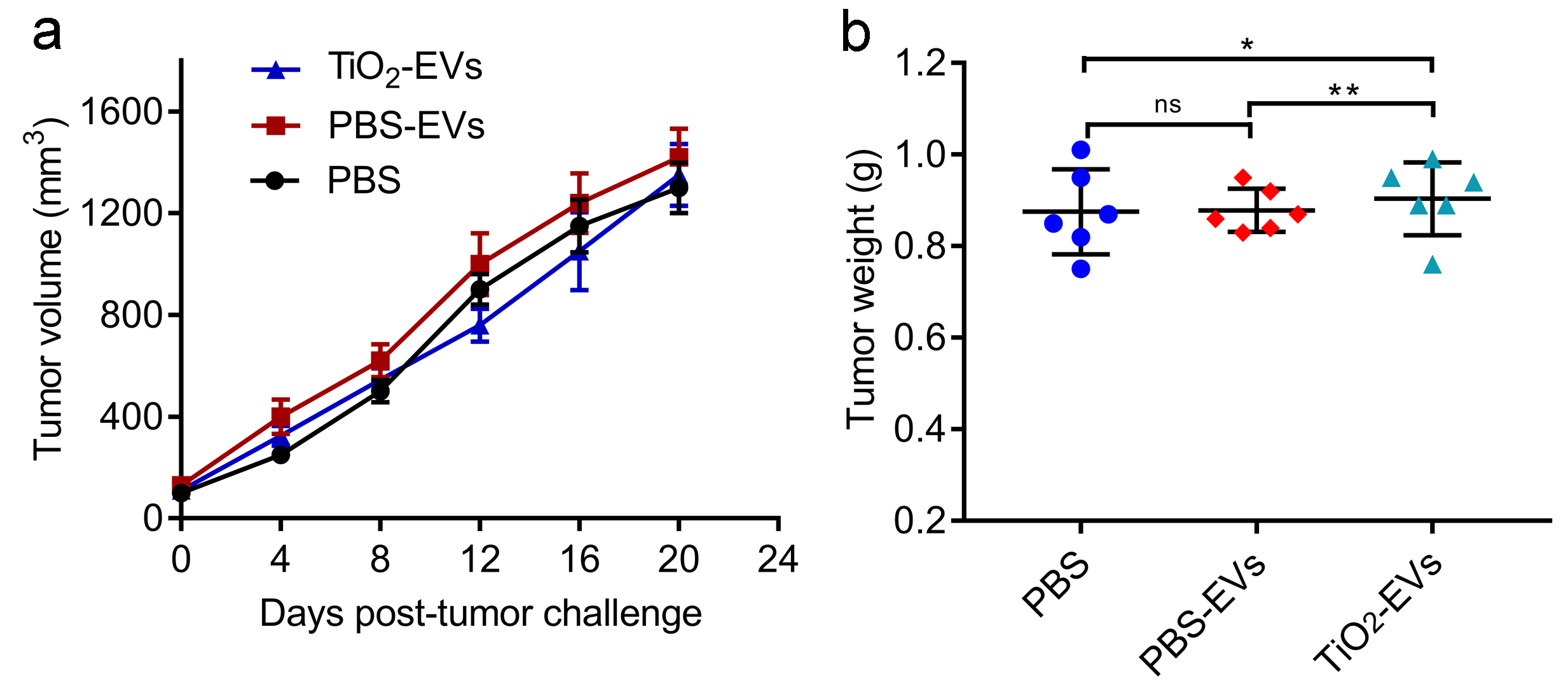


**Fig.S3** Tumor volume **(a)** and tumor weight **(b)** of mice treated with PBS-EVs or TiO_2_-EVs during the period of 21 days post-tumor challenge. Data are presented as mean value ± SD (n=6). Statistical analysis was measured by one-way ANOVA.

**
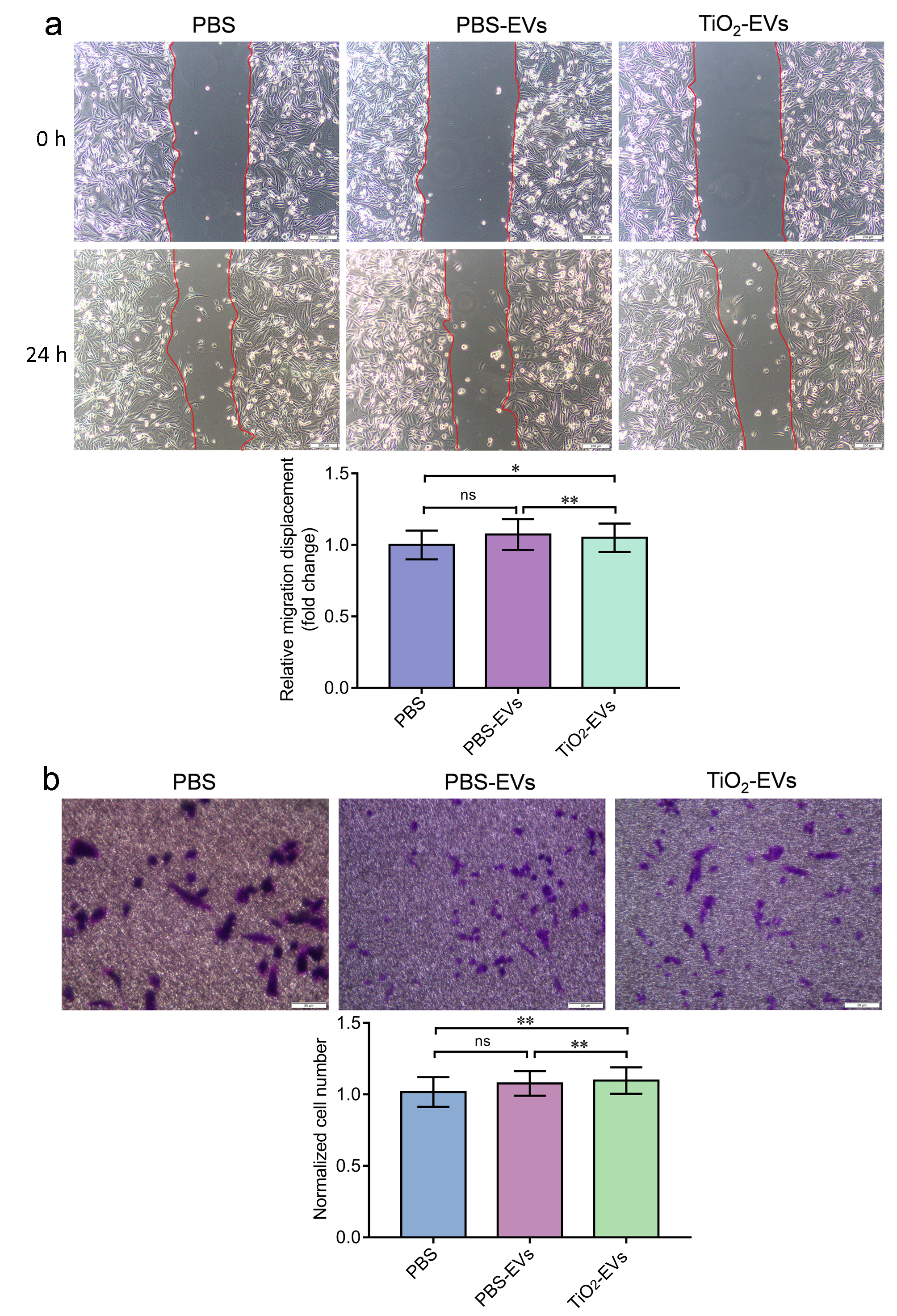
**

**Fig. S4** The effects of PBS-EVs or TiO_2_-EVs on tumor migration ability. Wound healing assay **(a)** and transwell assay **(b)** were used to detect the effects of PBS-EVs or TiO_2_-EVs on tumor migration ability. Scale bars =50 μm. Data are presented as mean value ± SD (n=3). Statistical analysis was measured by one-way ANOVA.


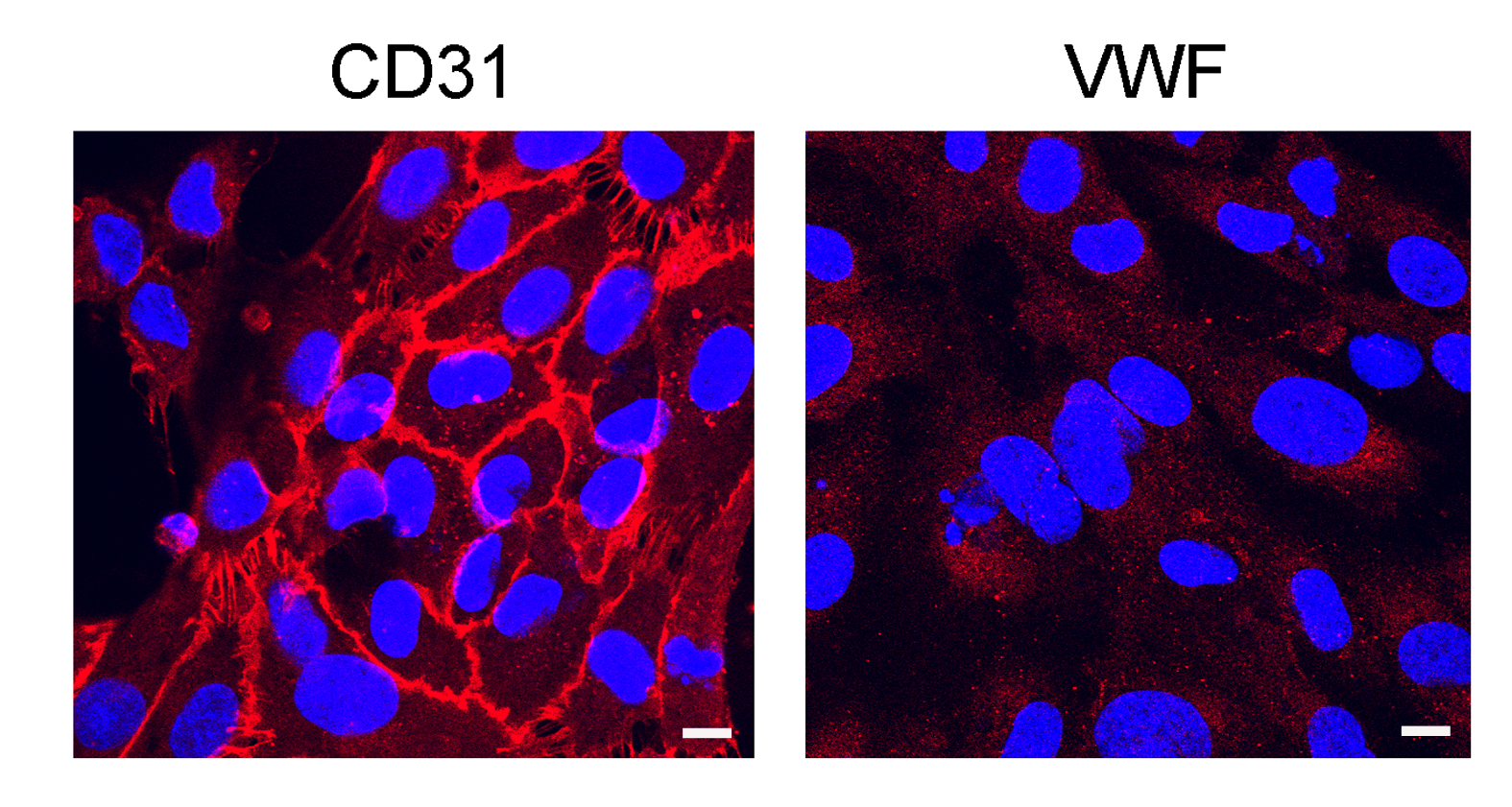


**Fig. S5** Immunofluorescence analysis of the expression of Von Willebrand factor (VWF) and platelet endothelial cell adhesion molecule-1 (CD31) in separated HUVECs. Scale bars =20 μm. Nuclei, Blue (DAPI); CD31 or VWF, Red.

**
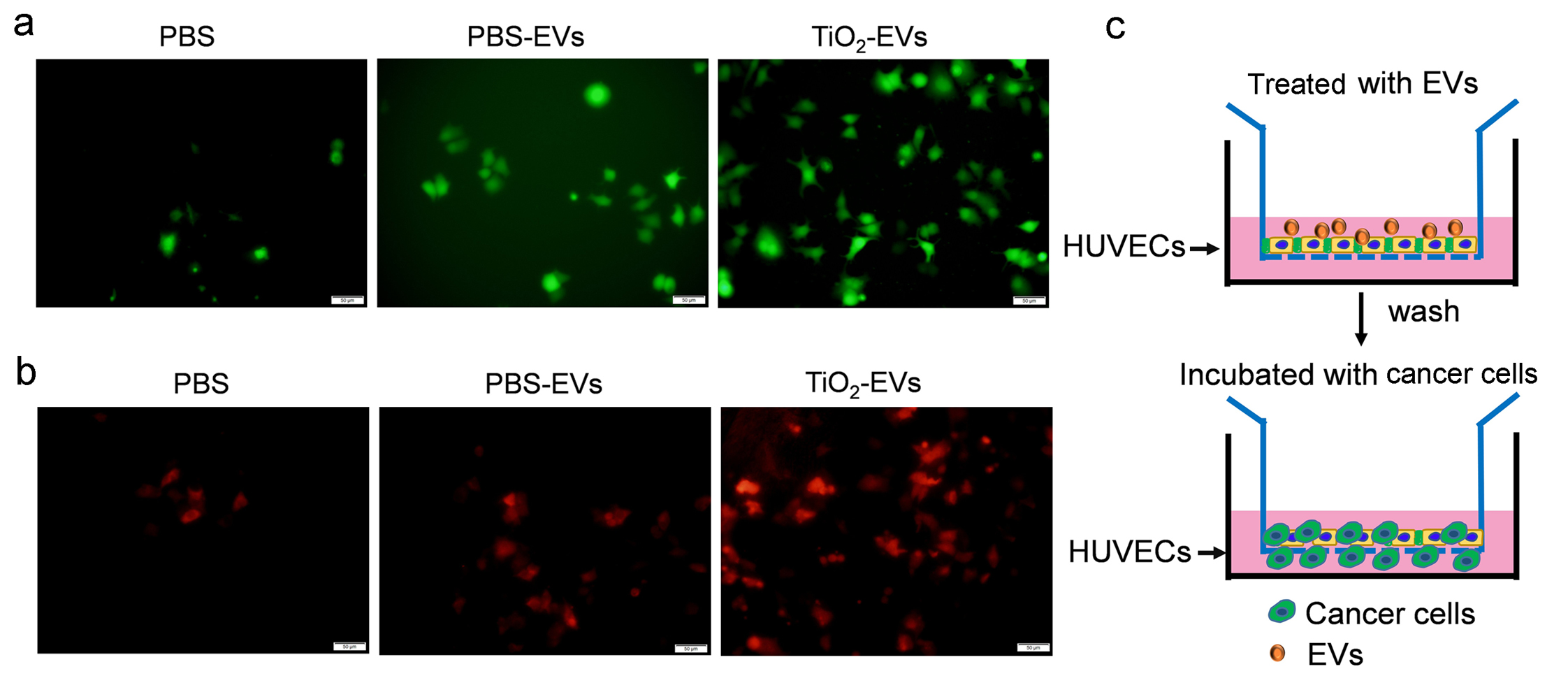
**

**Fig. S6** Cell-Tracker Green labeled MCF-7 cells (**a**) and Cell-Tracker Red labeled A549 cells (**b**) migrated through HUVECs layers were observed under fluorescent microscopy following exposure of HUVECs to EVs for 1 h. Scale bars =50 μm. (**c**) Experimental scheme for the migration assay.

**
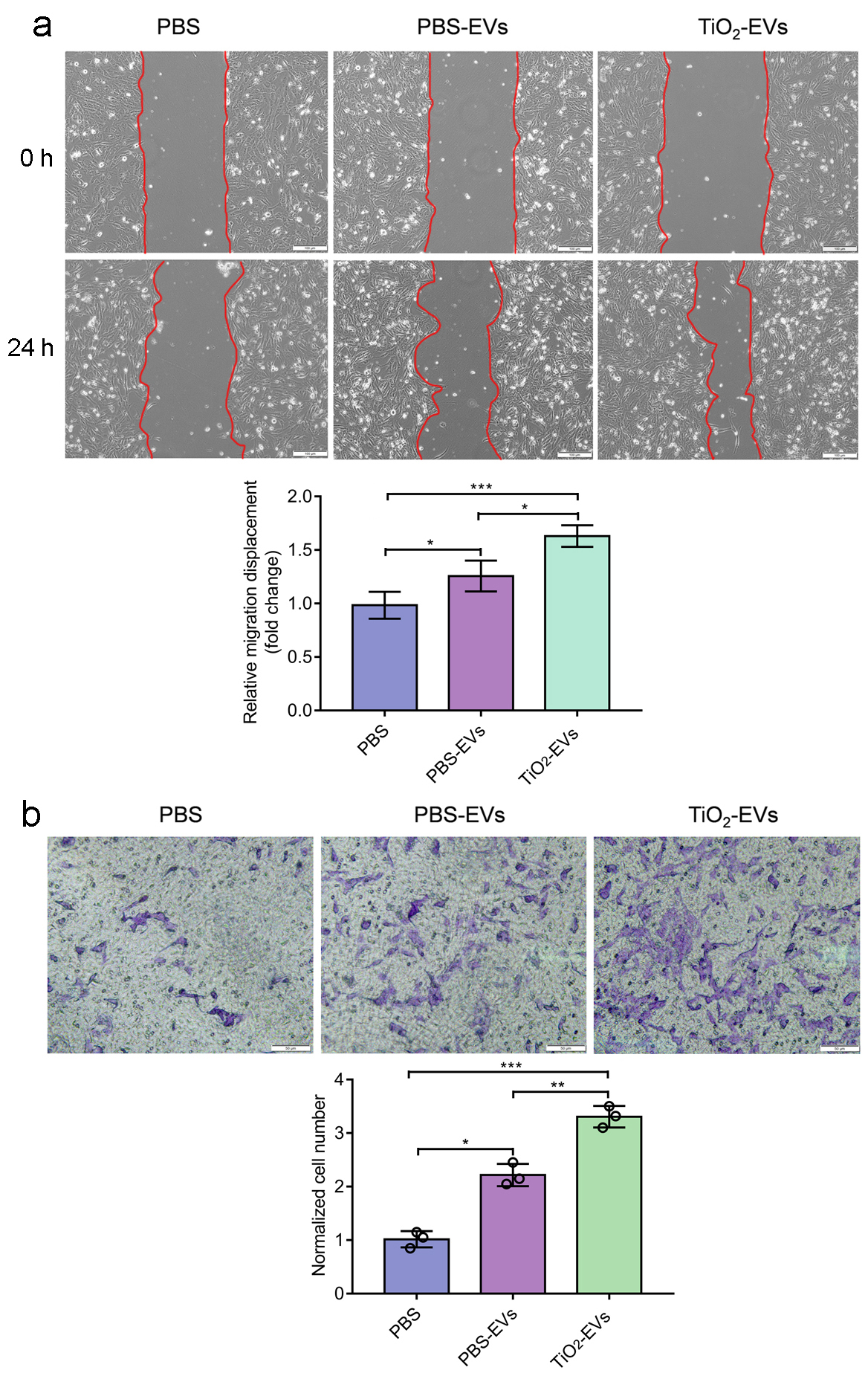
**

**Fig. S7** The effects of PBS-EVs or TiO_2_-EVs on HUVECs migration ability. Wound healing assay **(a)** and transwell assay **(b)** were used to detect the effects of PBS-EVs or TiO_2_-EVs on HUVECs migration ability. Scale bars =50 μm. Data are presented as mean value ± SD (n=3). Statistical analysis was measured by one-way ANOVA.

**
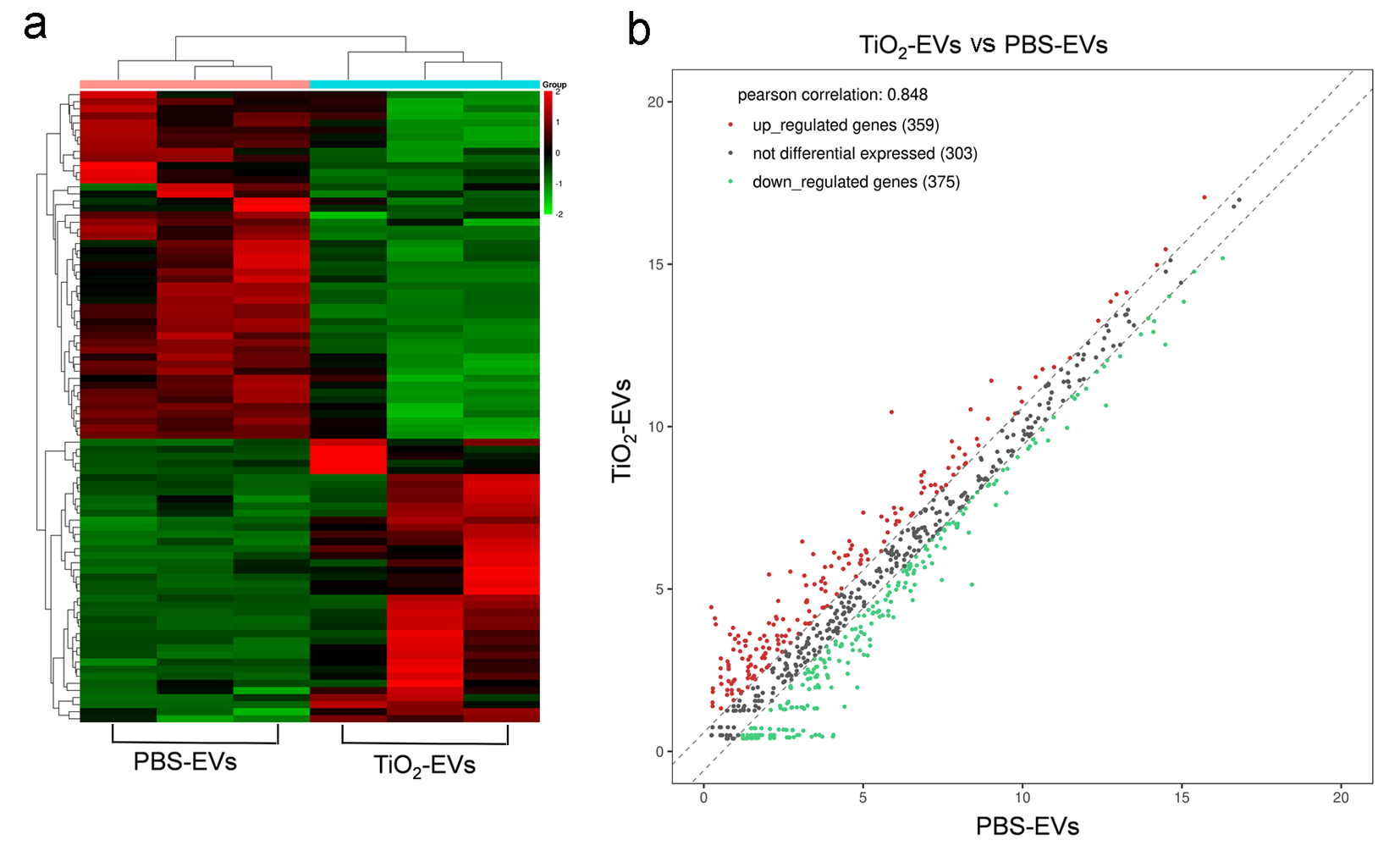
**

**Fig. S8** **(a)** Cluster diagram of differential expression miRNA. Each row represented one miRNA, and each column represented one sample. The red dots and green dots represented the upregulated and downregulated miRNAs, respectively **(b)** Scatter Plot of the DE-miRNAs. The black dots represent miRNAs that are not differentially expressed between PBS-EVs and TiO_2_-EVs, and the red dots and green dots represent the upregulated and downregulated miRNAs, respectively.


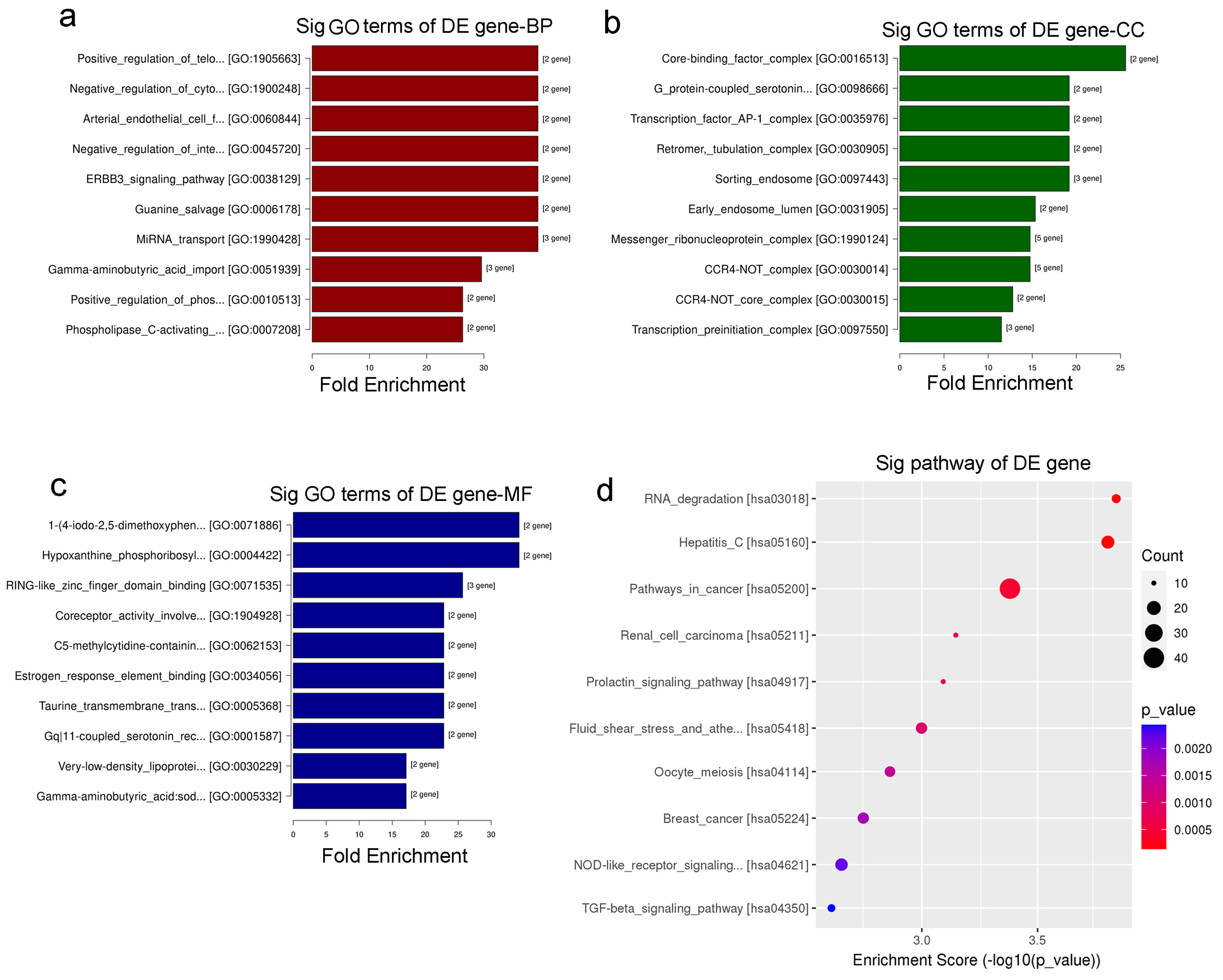


**Fig. S9** GO functions for the potential target genes of the top upregulated miRNAs were performed, including **(a)** Biological process (BP), **(b)** Cellular component (CC), **(c)** Molecular function (MF). **(d)** KEGG signaling pathway enrichment analysis were performed for significant differences in cancer related genes between PBS-EVs and TiO_2_-EVs.


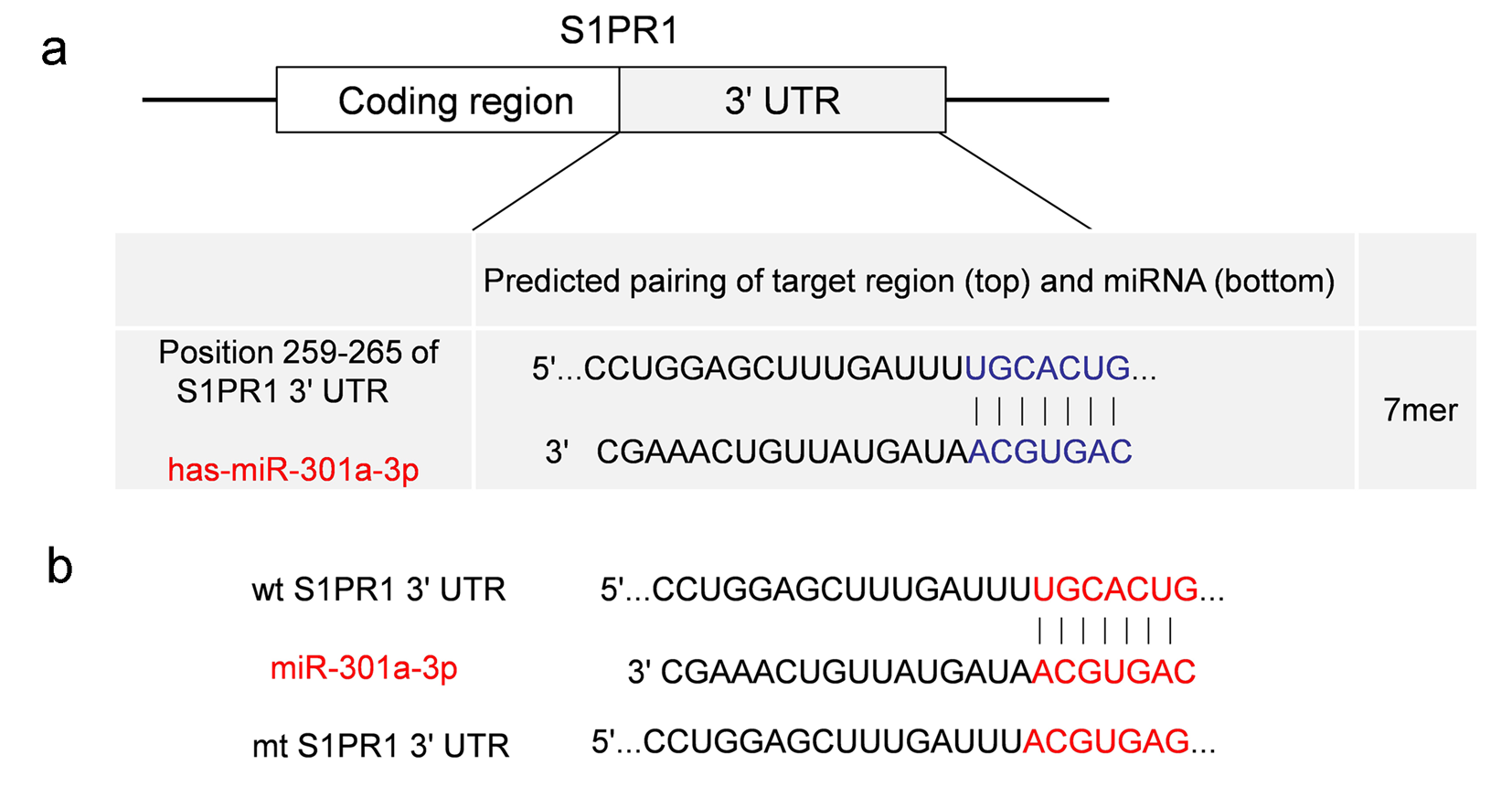


**Fig. S10 (a)** Prediction of the binding sites of miR-301a-3p and 3′-UTR of S1PR1. **(b)** Construction of a reporter gene plasmid containing wild-type (wt) or mutant (mt) 3′-UTR of S1PR1 binding sites.


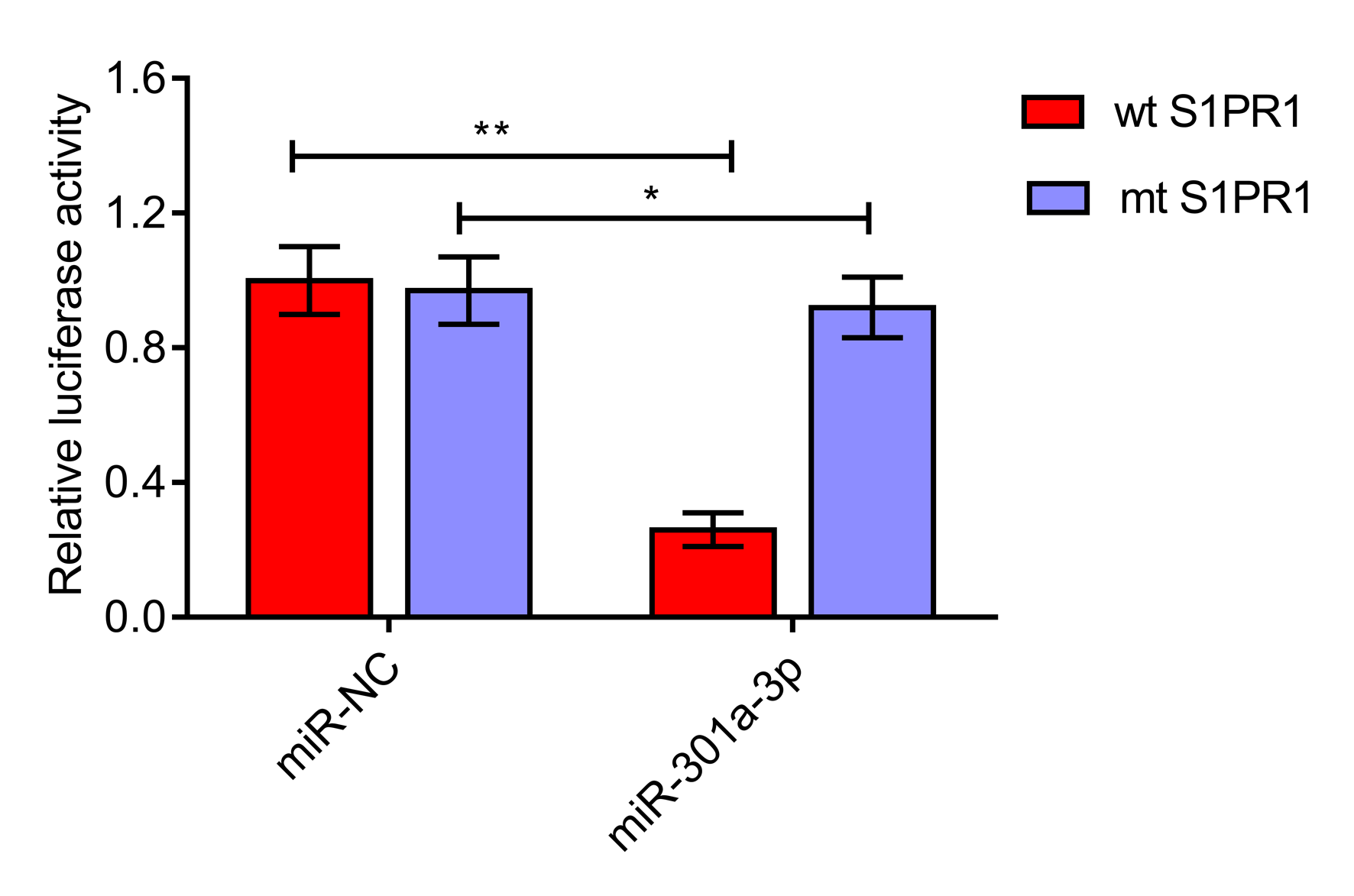


**Fig. S11** The relative luciferase activity of wild-type (wt) and mutant-type (mt) 3′-UTR of S1PR1 was determined in HUVECs. miR-301a-3p inhibited the luciferase activity of reporter containing wild-type but not mutant 3′-UTR of S1PR1. Data are shown as mean ± SD (n=3). Statistical analysis was measured by one-way ANOVA.


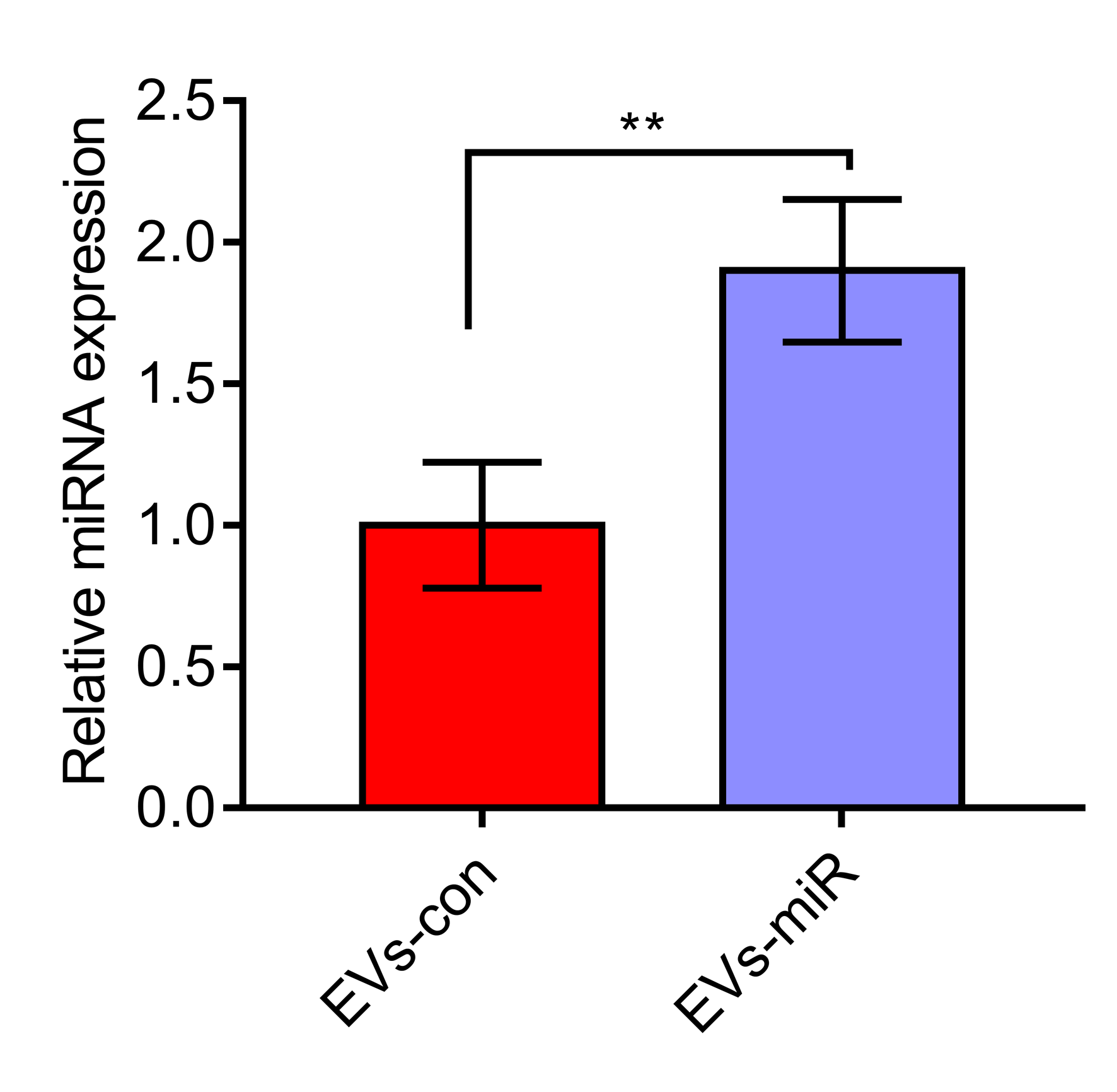


**Fig. S12** RT-PCR analysis of miR-301a-3p expression in EVs derived from MDA-MB-231 cells which were treated with a miR-301a-3p mimic. Data are shown as mean ± SD (n=3). Statistical analysis was measured by one-way ANOVA.


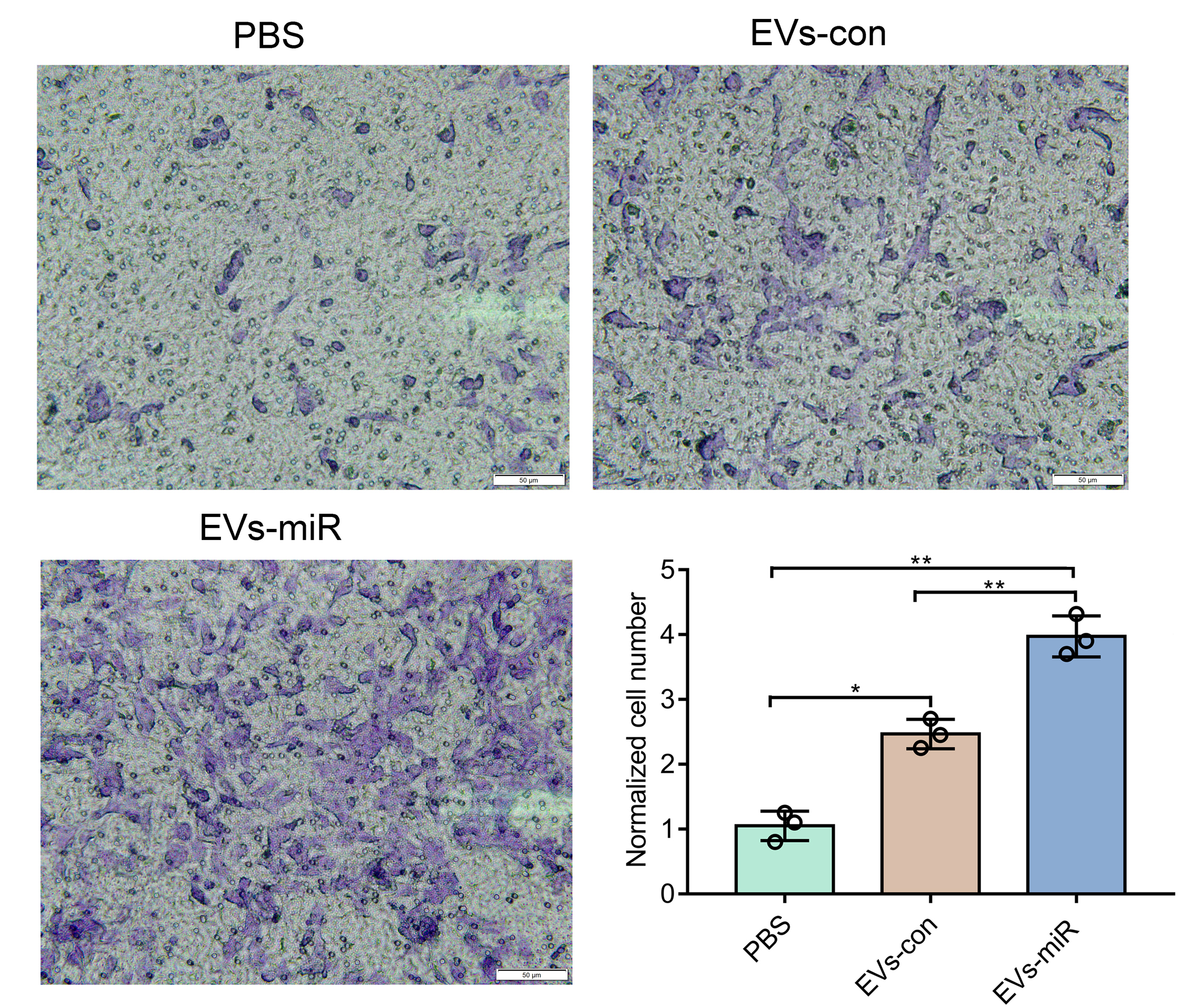


**Fig. S13** Transwell assay were used to detect the effects of EVs-con or EVs-miR on HUVECs migration ability. Scale bars =50 μm. Data are presented as mean value ± SD (n=3). Statistical analysis was measured by one-way ANOVA.


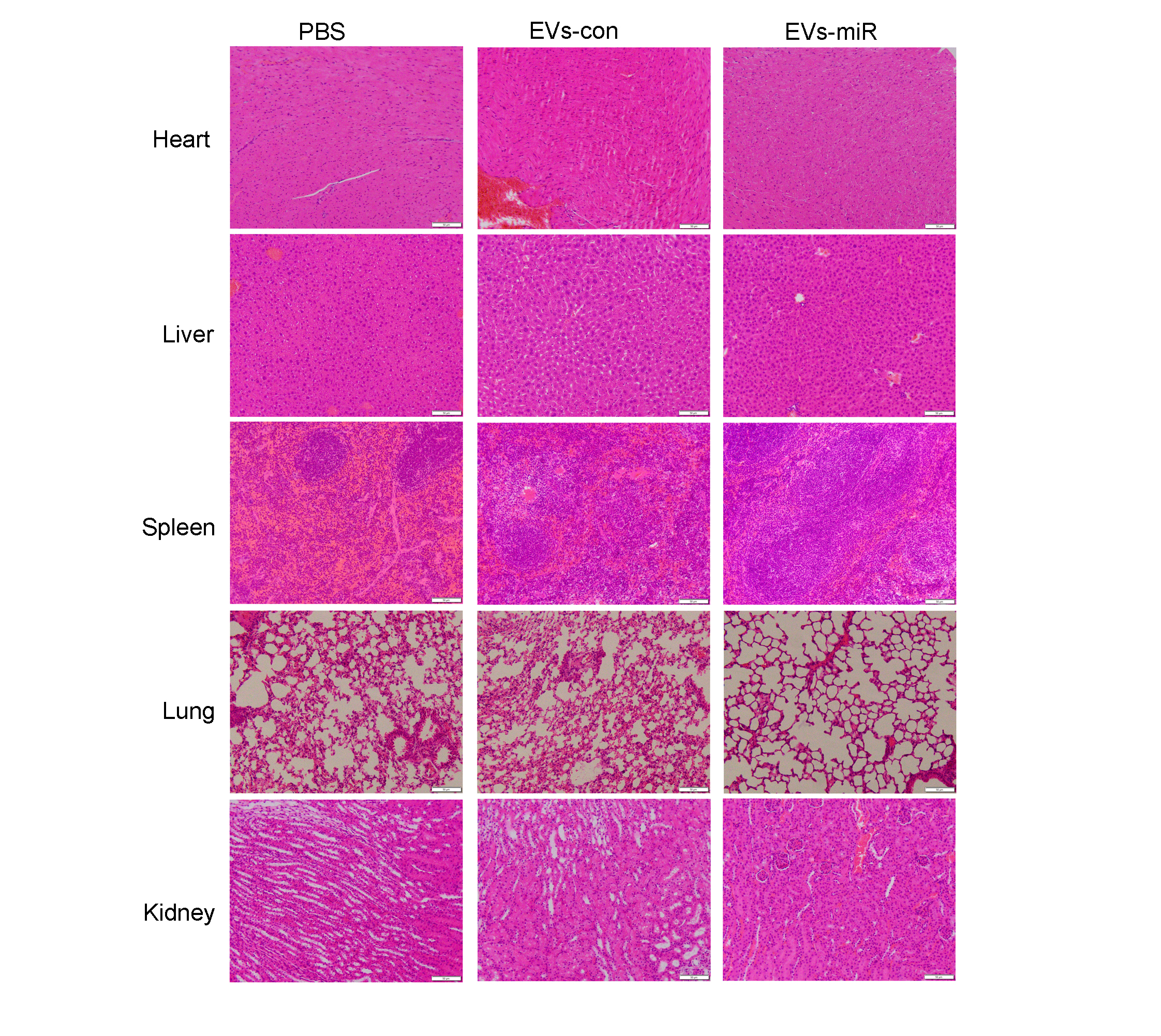


**Fig. S14** HE staining images of the major organs of mice from different treatment groups. The results indicated that the EVs had no obvious toxic effects on the organs of mice. Scale bar =50 µm.

**Table S1** The primers used in the mRNA/miRNA expression analysis experiment.

| Primer name | Sequence (5ʹ-3ʹ) |
| --- | --- |
| U6 | F: CTCGCTTCGGCAGCACA  R: AACGCTTCACGAATTTGCGT |
| miR-301a-3p | F:ACACTCCAGCTGGGCAGTGCAATAGTATTGTC  R: CTCAACTGGTGTCGTGGA |
| GAPDH | F: ATGGAAATCCCATCACCATCTT  R: CGCCCCACTTGATTTTGG |
| S1PR1 | F: AGCGTTTGTCTGGAGAAGTACC  R: TAGCAAGGAGGCTGAAGACTGA |
| VEGFR2  miR-555 | F: TCATTATTCTAGTAGGCACGGCG  R: GACAAGTAGCCTGTCTTCAGTT  R: GTGCAGGGTCCGAGGT  F: CCGTAAGCTGAACCTCTGATAA |
| miR-653-5p  miR‑3195 | F:TTGAAACATTCTCTACTGAAC  R: GAACATGTCTGCGTATCTC  F: GCGCCGGGCCCGGGTT  R: ACTCCCATGCCAAGAACTCC |
| miR-500a-5p  miR-556-3p  miR-570-3p  miR-3143  miR-429  miR-501-5p  miR-532-3p  miR‑301a‑3p mimic | F:ACACTCCAGCTGGGAUGCACCUGGGCA  R: TGGTGTCGTGGAGTCG  F: ATATTACCATTAGCTCATCTTT  R: TGGTGTCGTGGAGTCG  F:ACACTCCAGCTGGGCGAAAACAGCAA  R: TGGTGTCGTGGAGTCG  F:GCCGAGATAACATTGTAAAGCGC  R: CTCAACTGGTGTCGTGGA  F:GCCGAGTAAUACTGTCTGGT  R: CTCAACTGGTGTCGTGGA  F: AUCCUUUGUCCCUGGGUGAGA  R: TGGTGTCGTGGAGTCG  F: ATCCTCCCACACCCAAGG  R: GTGCAGGGTCCGAGGT  GCUCUGACUUUAUUG CACUACU |
| NC mimic | UCACAACCUCCUAGAAAGAGUAGA |
| miR-301a‑3p inhibitor | AGUAGUGCAAUAAAGUCAGAGC |
| NC inhibitor | UCUACUCUUUCUAGGAGGUUGUGA |

**Fig. S15** The membrane images for all Western blotting reported in the main figures of this study.

Fig. 3e


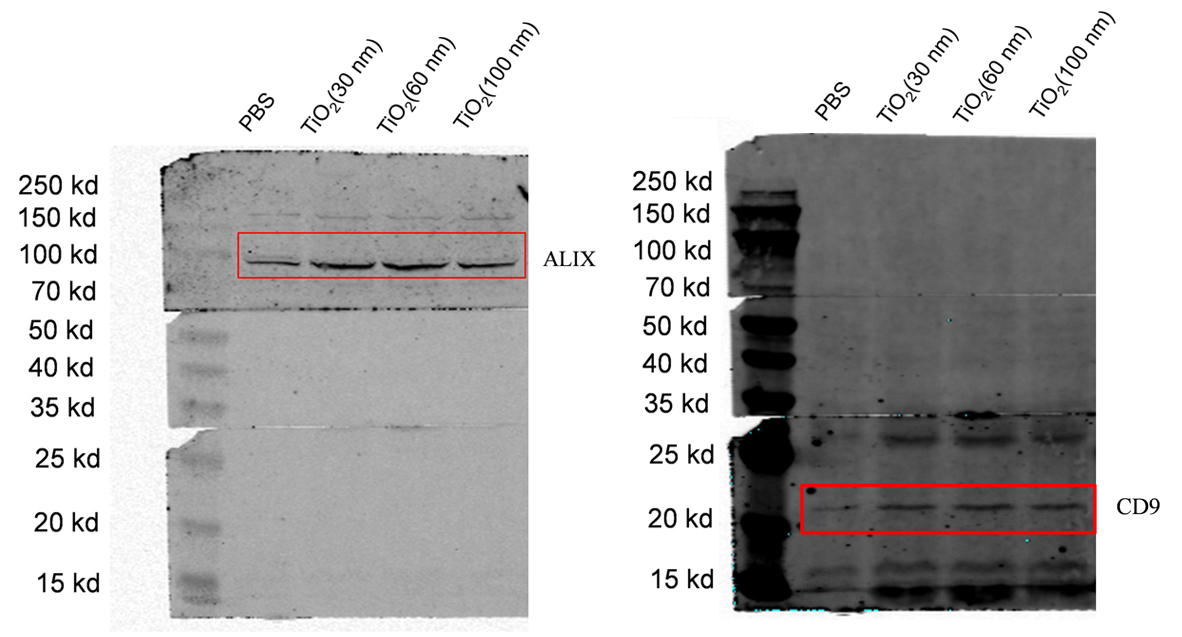


Fig. 3f


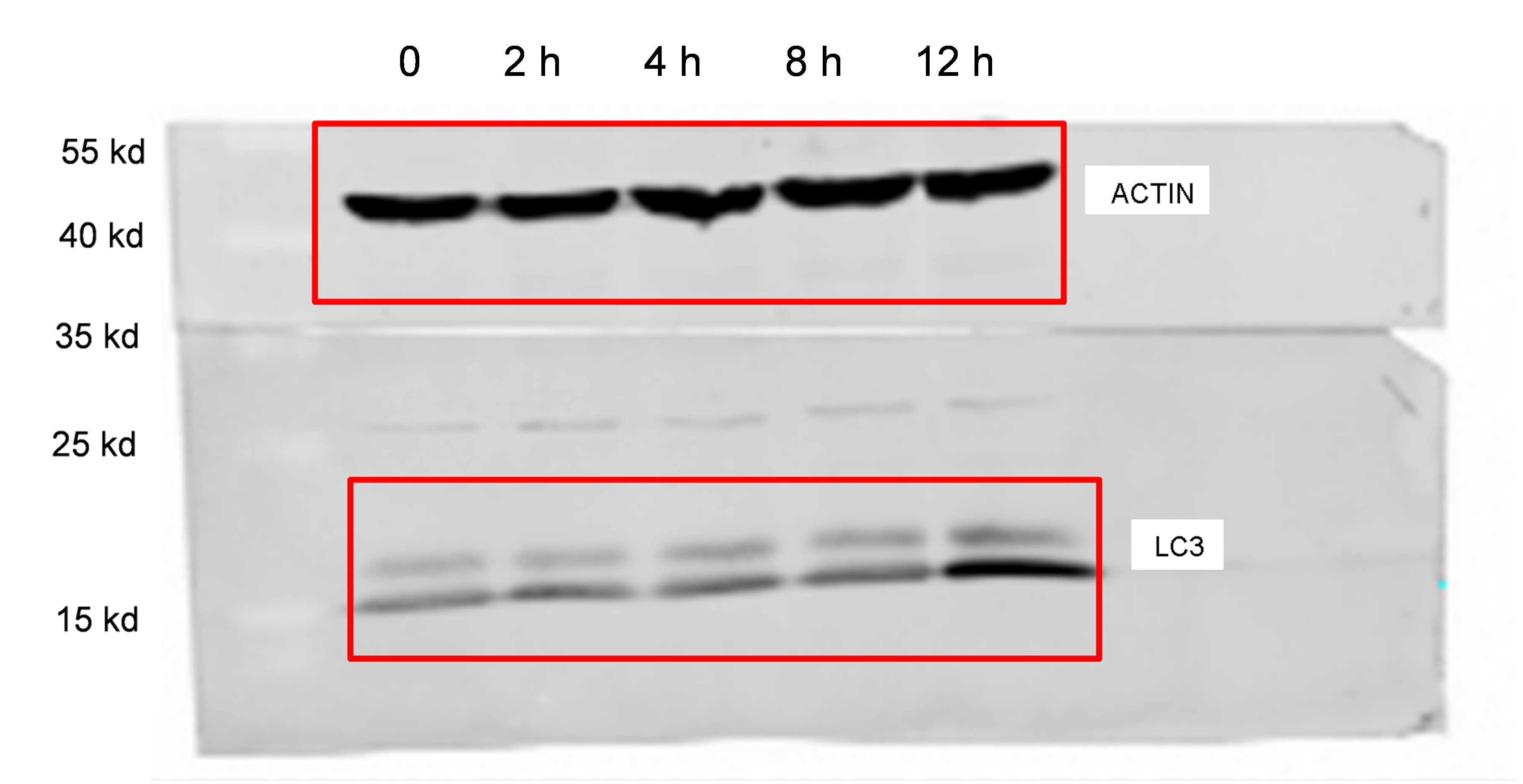


Fig. 3h


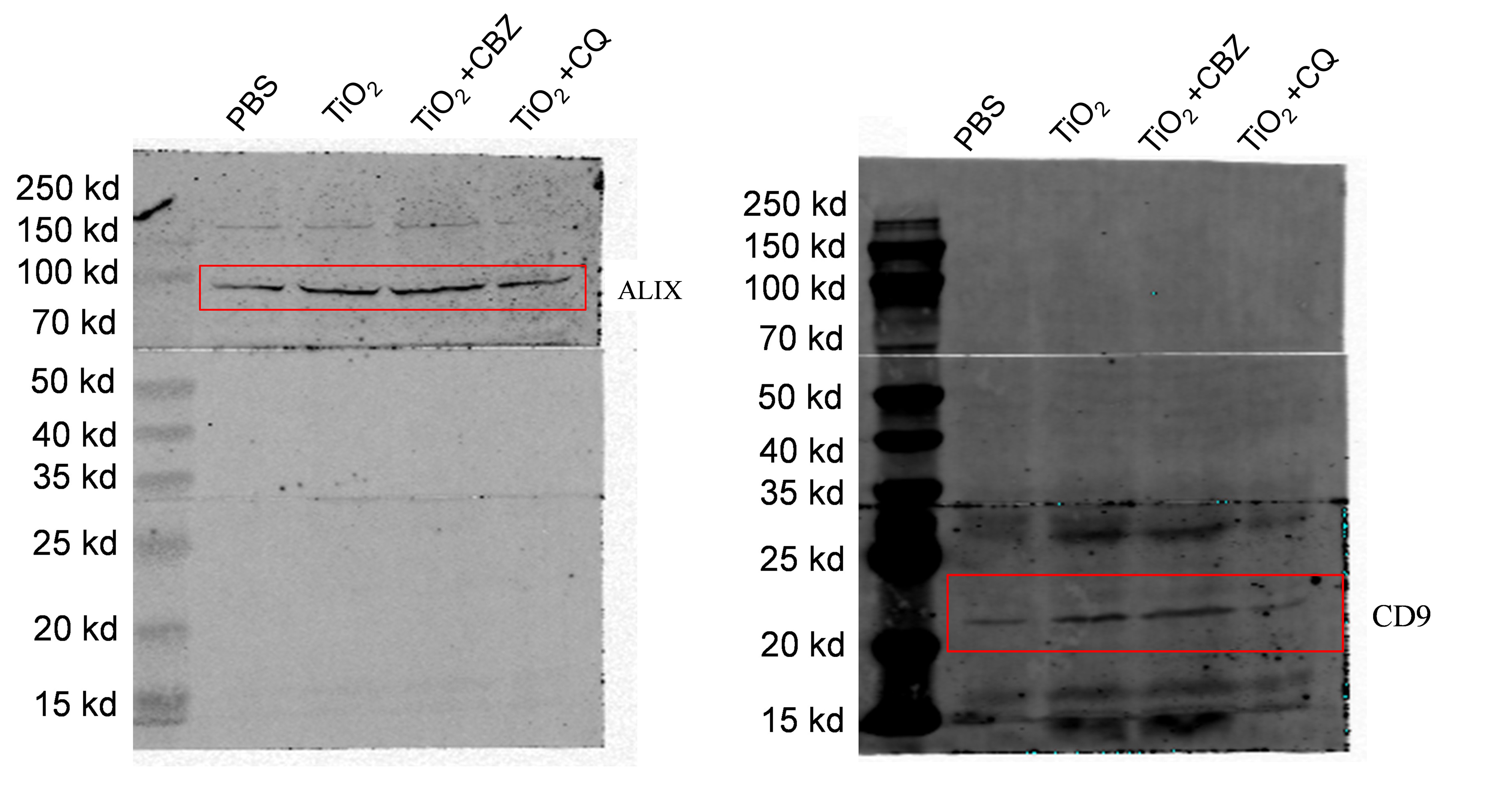


Fig. 4h


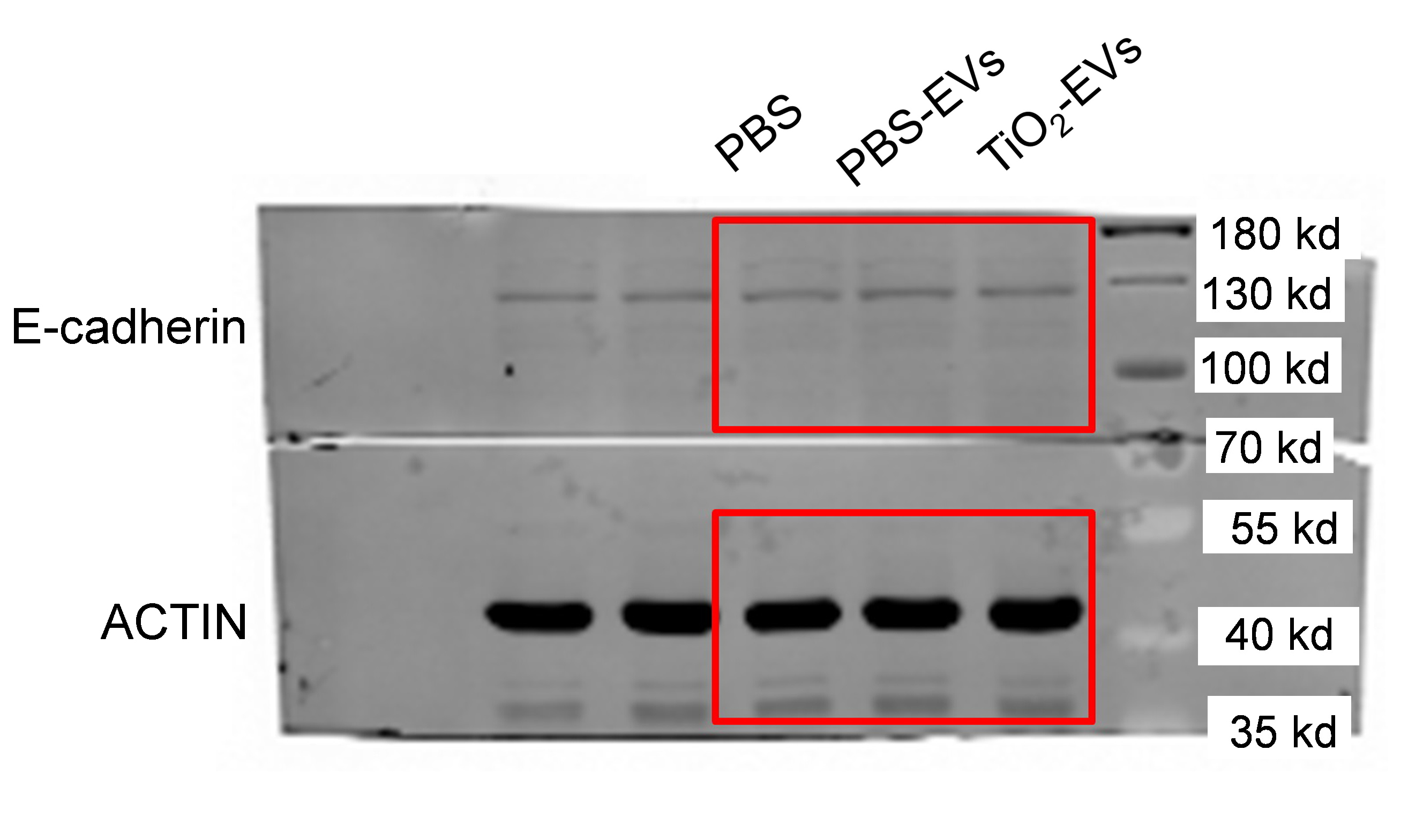


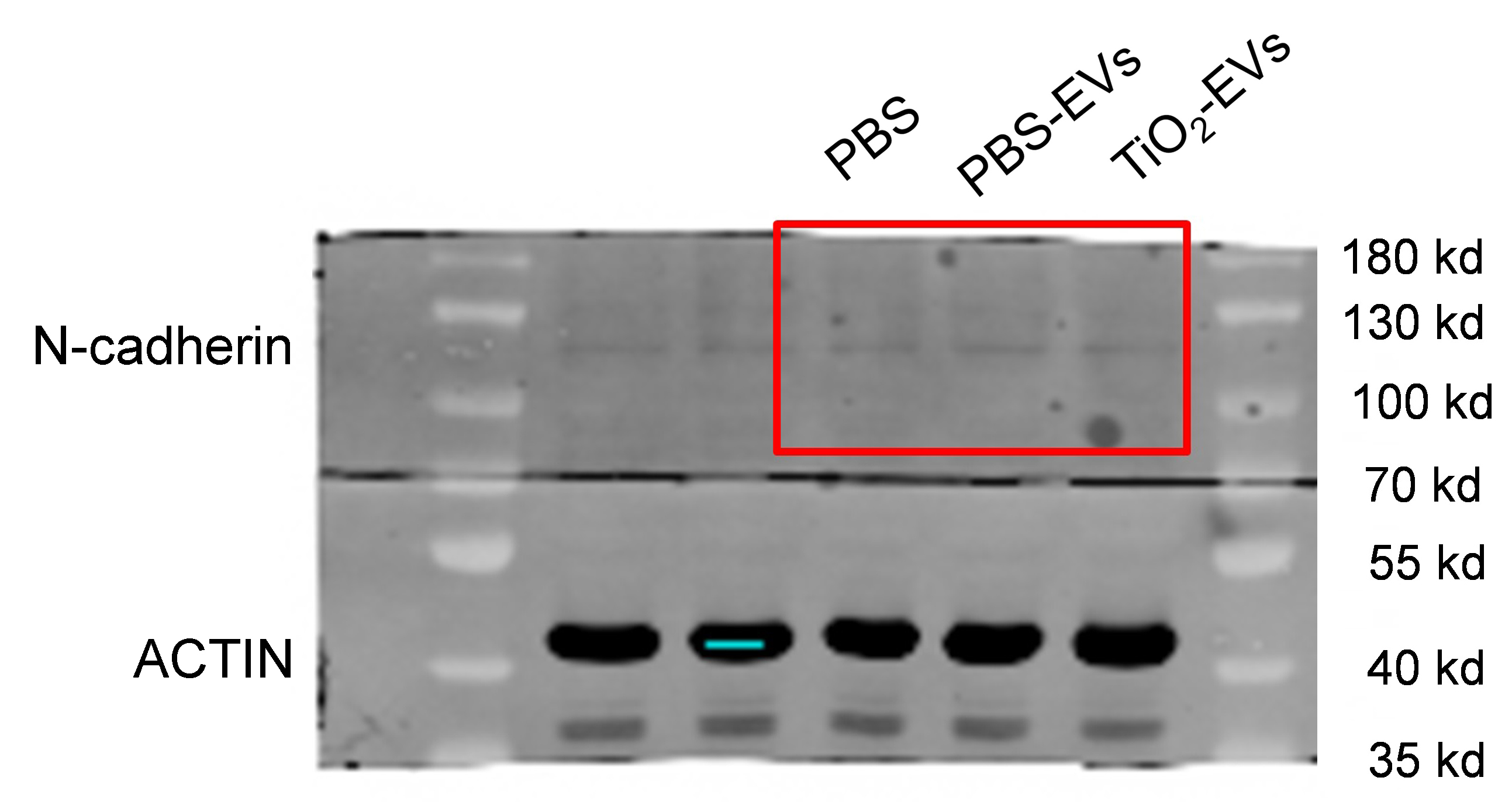


Fig. 5e


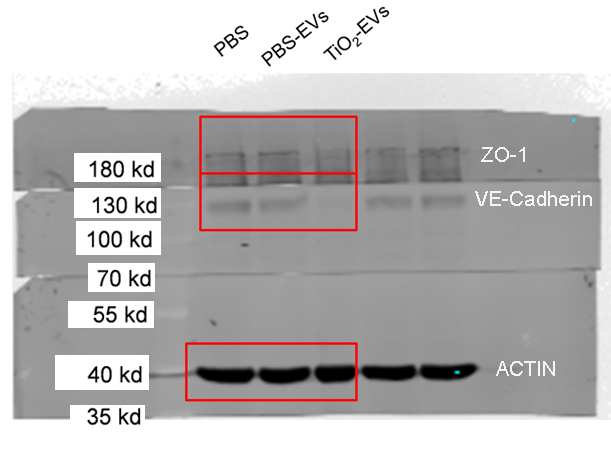


Fig. 6e


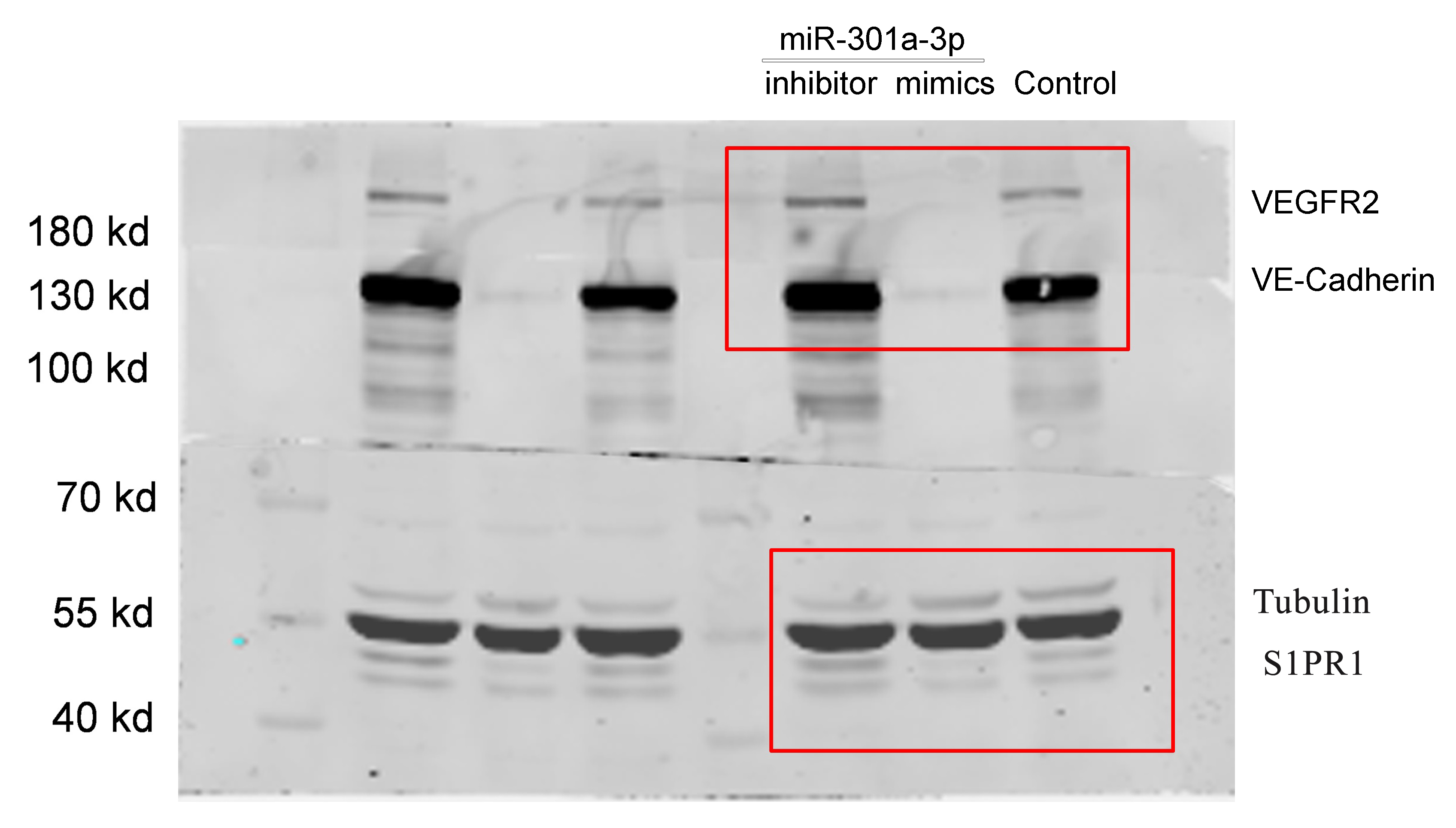

Supplement: Supplementary file 1 — Additional file 1: Figure S1. TiO2 inorganic NPs promoted tumor metastasis in vivo. a Body weight of mice treated with PBS or TiO2 during the period of 21 days post-tumor challenge. b Tumor weight of mice treated with PBS or TiO2 during the period of 21 days post-tumor challenge. Data are presented as mean value ± SD (n = 6). Statistical analysis was measured by one-way ANOVA. Figure S2. TEM images of TiO2 NPs with different sizes. a 30 nm, b 60 nm. Scale bars = 100 nm. c 100 nm. Scale bars = 200 nm. Figure S3. Tumor volume (a) and tumor weight (b) of mice treated with PBS-EVs or TiO2-EVs during the period of 21 days post-tumor challenge. Data are presented as mean value ± SD (n = 6). Statistical analysis was measured by one-way ANOVA. Figure S4. The effects of PBS-EVs or TiO2-EVs on tumor migration ability. Wound healing assay (a) and transwell assay (b) were used to detect the effects of PBS-EVs or TiO2-EVs on tumor migration ability. Scale bars = 50 μm. Data are presented as mean value ± SD (n = 3). Statistical analysis was measured by one-way ANOVA. Figure S5. Immunofluorescence analysis of the expression of Von Willebrand factor (VWF) and platelet endothelial cell adhesion molecule-1 (CD31) in separated HUVECs. Scale bars = 20 μm. Nuclei, Blue (DAPI); CD31 or VWF, Red. Figure S6. Cell-Tracker Green labeled MCF-7 cells (a) and Cell-Tracker Red labeled A549 cells (b) migrated through HUVECs layers were observed under fluorescent microscopy following exposure of HUVECs to EVs for 1 h. Scale bars = 50 μm. c Experimental scheme for the migration assay. Figure S7. The effects of PBS-EVs or TiO2-EVs on HUVECs migration ability. Wound healing assay (a) and transwell assay (b) were used to detect the effects of PBS-EVs or TiO2-EVs on HUVECs migration ability. Scale bars = 50 μm. Data are presented as mean value ± SD (n = 3). Statistical analysis was measured by one-way ANOVA. Figure S8. a Cluster diagram of differential expression miRNA. Each row represented one miRNA, [file 12951_2023_2142_MOESM1_ESM.doc]
